# Strontium Isotopes and the Reconstruction of the Chaco Regional System: Evaluating Uncertainty with Bayesian Mixing Models

**DOI:** 10.1371/journal.pone.0095580

**Published:** 2014-05-22

**Authors:** Brandon Lee Drake, Wirt H. Wills, Marian I. Hamilton, Wetherbee Dorshow

**Affiliations:** Department of Anthropology, University of New Mexico, Albuquerque, New Mexico, United States of America; New York State Museum, United States of America

## Abstract

Strontium isotope sourcing has become a common and useful method for assigning sources to archaeological artifacts. In Chaco Canyon, an Ancestral Pueblo regional center in New Mexico, previous studies using these methods have suggested that significant portion of maize and wood originate in the Chuska Mountains region, 75 km to the East. In the present manuscript, these results were tested using both frequentist methods (to determine if geochemical sources can truly be differentiated) and Bayesian methods (to address uncertainty in geochemical source attribution). It was found that Chaco Canyon and the Chuska Mountain region are not easily distinguishable based on radiogenic strontium isotope values. The strontium profiles of many geochemical sources in the region overlap, making it difficult to definitively identify any one particular geochemical source for the canyon's pre-historic maize. Bayesian mixing models support the argument that some spruce and fir wood originated in the San Mateo Mountains, but that this cannot explain all ^87^Sr/^86^Sr values in Chaco timber. Overall radiogenic strontium isotope data do not clearly identify a single major geochemical source for maize, ponderosa, and most spruce/fir timber. As such, the degree to which Chaco Canyon relied upon outside support for both food and construction material is still ambiguous.

## Introduction

**Figure 1:**
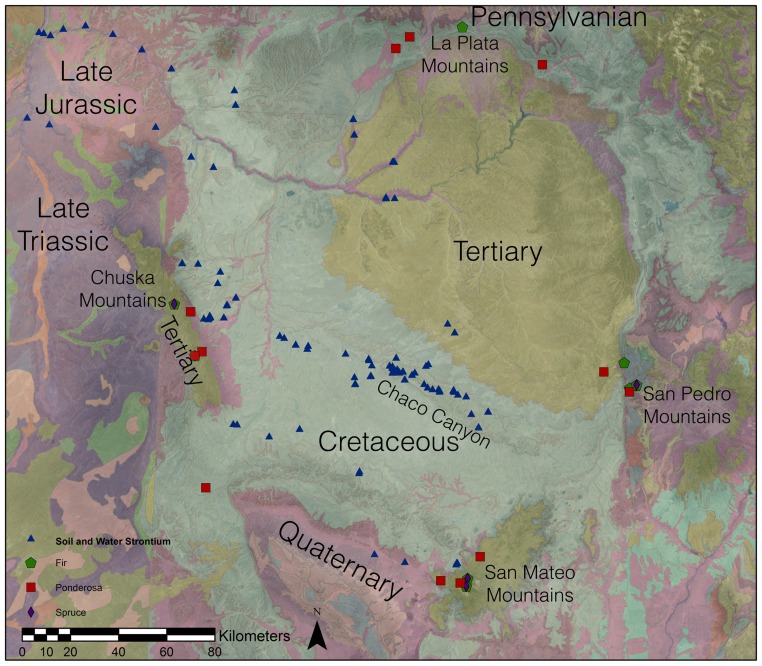
Location of Chaco Canyon (center point) in the San Juan Basin, surrounding upland areas and location of strontium samples associated with forested zones. A simplified geology (colored based on age) shows the relation of strontium sampling locations to their parent geology.

**Figure 2:**
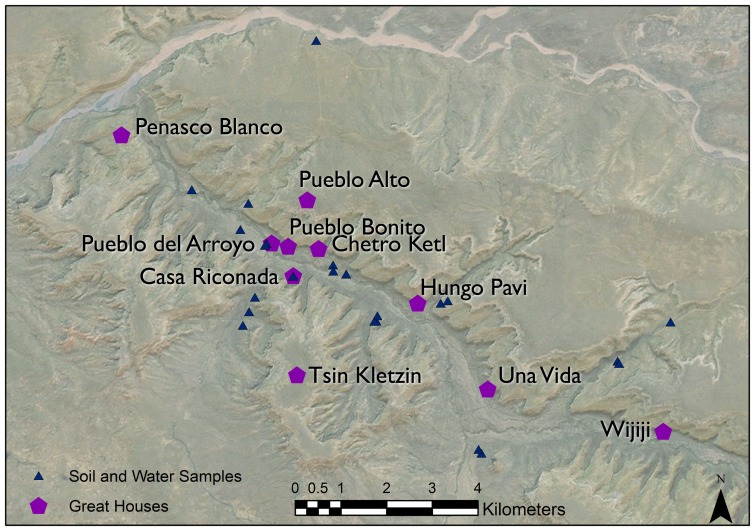
Chaco Canyon Great Houses, local tributaries and strontium sample locations associated with maize sourcing studies [9].

During the Bonito Phase (ca. AD 860 to 1140) in Chaco Canyon, New Mexico (Figure 1), a series of dispersed subsistence farming communities coalesced around the construction of communal stone buildings called “Great Houses,” of which Pueblo Bonito is the most famous (Figure 2). Often referred to as the “Chaco Phenomenon,” the social changes that occurred during the Bonito Phase are widely viewed as characteristic of emergent social complexity. Bonito Phase food production was based on the cultivation of maize, beans and squash, and possibly non-domesticates such as sunflowers, and there is evidence for water control features designed to direct seasonal runoff into fields [1]. These crops were grown in Chaco despite constraints imposed by aridity and high elevation, but there is considerable debate among specialists about whether the canyon could have produced enough food to support “monumental” Great House construction efforts or even sustain a relatively small residential population [2]. If the local economy could not underwrite the labor requirements of construction or meet the basic physiological needs of a small canyon residential community, then an obvious conclusion is that labor and food were brought into Chaco from somewhere else [3]. The Bonito Phase is famous for evidence of long-distance exchange in materials, especially pottery, turquoise, macaws, cacao, and timber [4]–[6] and therefore it is reasonable to think that food could have moved through these same exchange systems.

Over the past decade a series of studies based on strontium isotope analysis of maize (*Zea mays*) have attempted to identify specific source areas (farming locations) outside of Chaco [2] [7]–[10]. Researchers have concluded that at least some maize was imported from distances of up to 75 km. Diamond (2005) has argued that reliance on such long-distance exchange networks was an unsustainable economic strategy that set up conditions for the rapid collapse and abandonment of Chaco society during a major 12th century drought [10]. Emergent complex societies in the past are typically associated with evidence for long-distance exchange [11]; the demonstration of long-distance transport for construction materials and food would imply a high level of complexity in Chaco Canyon [12]. General conditions may have been wetter at the time [13], with Great House construction occurring during wet periods [14].

However, the following study illustrates that radiogenic strontium isotope (^87^Sr/^86^Sr) values obtained from Chaco maize do not discriminate statistically between the ^87^Sr/^86^Sr values of purported distant geographical sources and ^87^Sr/^86^Sr values of sources within Chaco Canyon itself [7]. A second set of strontium isotope analyses designed to identify possible long-distance sources of timber used to build Great Houses [12] [15] showed a similar lack of clear discrimination among potential sources of ponderosa pine (*Pinus ponderosa*), the primary construction species. These same studies, however, indicated potential timber sources in the canyon drainage system. Building on recent models showing that the prehistoric agricultural potential of the canyon has been previously underestimated [16]–[17], it is suggested here that the importation of costly basic resources during the Bonito Phase has not yet been proven, despite popular assumptions that Great House society was based on such transactions [10].

### Background

Scholars propose that in the 11^th^ century AD, Chaco saw the development of a meta-community of Great Houses as well as dozens of smaller residential buildings hierarchically organized as a way to effectively manage social relations, food production, and regional exchange [18]–[25]. In turn, the concentration of Great Houses in the canyon (Figure 2) is usually seen as the sociopolitical center of a larger regional network of farming communities outside Chaco, each containing at least one Great House [26]. Most Great House architecture in Chaco was built during the 11^th^ century A.D., with construction declining precipitously after AD 1100, and ending by the late AD 1200s or earlier [27] [28].

The San Juan basin is a mixture of Pennsylvanian, Triassic, Jurassic, Cretaceous, Tertiary, and Quaternary sedimentary formations (Figure 1, Figure S1, S2, S3) [29]. Today, Chaco Canyon itself holds few advantages for dry-farming. The canyon's high elevation (1800 to 1900 masl), aridity, b-seasonal precipitation, and cold winters make cultivation a risky endeavor. Paleoenvironmental studies suggest that the canyon vegetation has changed little over the past 2000 years, leading many researchers to speculate that food was brought into the canyon from elsewhere. Studies in the possible sources of construction beams and maize were spurred on by the recognition that the major mountain ranges near the Colorado Plateau had differing geochemistries [12], chief among these being radiogenic strontium isotope ratios. Evidence for a substantial presence of spruce and fir in Chaco Canyon during the period of construction of the Great Houses is limited [30]–[33]. The long distance transport of tree beams had been hypothesized before [34] [35], and ^87^Sr/^86^Sr values could serve as valid test. Strontium atoms themselves have the same outer electron valence shell structure as calcium, and can be substituted for calcium in plant and animal tissues. Radiogenic (^87^Sr) to non-radiogenic (^86^Sr) strontium isotope ratios are unique across many geological terrains, and therefore measuring them can be useful in sourcing various materials. Radiogenic strontium (^87^Sr) forms as radioactive rubidium decays. The amount of ^87^Sr in any particular substrate is a function of the age of the rock as well as the original abundance of strontium and rubidium. When set as a ratio with non-radiogenic strontium, variation in abundance is normalized [36]. Bedrock weathering is the primary input for soil ^87^Sr/^86^Sr [36] [37] but alluvial and aeolian deposition can also influence ratios, along with differential weathering of minerals. However, differences between the radiogenic strontium isotope ratios of bedrock and soil are generally minimal [36] [38], though the ratio does vary by depth [39]. Plants acquire the ^87^Sr/^86^Sr value of the soils that they grow in, and numerous studies have confirmed that a plant's ^87^Sr/^86^Sr value is consistently a less variable, more averaged version of the local soil's [39] [40].

A notable contribution to this effort has been a long-term study examining the ^87^Sr/^86^Sr values from maize found in Chaco Canyon and its potential regional sources [2] [7] [8]. These studies analyzed synthetic soil-water samples collected from locations throughout the southern Colorado Plateau (including 48 in Chaco Canyon) and 36 maize cobs from nine Chaco sites, including seven from Pueblo Bonito. Twenty-one of the maize cobs from four sites all dated to the late 12^th^ century AD. The specimens from Pueblo Bonito ranged from the late 9^th^ to early 13^th^ centuries, while six samples from other sites dated to the 15^th^, 18^th^ and 19^th^ centuries ([7], Table 1). Although Benson and colleagues (2009) concluded that none of the analyzed maize came from sources in Chaco Canyon, they also recognized significant shortcomings in their comparative database stemming from geological complexity at the regional level. The geological variation observed by Benson's study group likely reflects the regional dominance of Cretaceous sandstone formations (Figure 1) combined with a variety of erosional and depositional processes (including aeolian mixing), all of which can lead to extremely patchy geochemical properties in potential cultivation sites.

**Table 1:** Comparison of medians across maize by provenience (columns) and potential sources (rows).

	Aztec Soil	Chuska Slope	Lobo Mesa	Northeastern San Juan River	Red Mesa	La Plata	Salmon Ruin	Pre 1140 Maize	Post 1140 Maize	Historic Maize
Chaco Watershed	−0.0003*	0.0002*	−0.0006**	0.0006**	0.0002	0.0004*	−0.0008**	**−0.0002**	−0.0009**	−0.0012**
Aztec Soil		0.0005**	−0.0003	0.0009**	0.0005*	0.0008*	−0.0005*	0.0001	−0.0006	−0.0009*
Chuska Slope			−0.0008**	0.0003**	0.0001	0.0002	−0.0011**	−0.0004**	−0.0011**	−0.00145**
Lobo Mesa				0.0011**	0.0008*	0.0010*	**−0.0003**	0.0004	−0.0003	**−0.0006**
Northwestern San Juan River					**−0.0003**	−0.0001	−0.0014*	−0.0008**	−0.0014**	−0.0018**
Red Mesa						0.0002	−0.0011*	**−0.0004**	−0.0011**	−0.0014*
La Plata							−0.0013*	−0.0006*	−0.0013**	−0.0016*
Salmon Ruin								**0.0006**	0	−0.0003
Pre 1140 Maize									**−0.0007**	**−0.0010**
Post 1140 Maize										−0.0003

Significance codes: 0.001 ‘**’ 0.01 ‘*’.

Items in bold indicate that no significant difference between medians was found.

Benson's efforts to identify geographic sources for maize found in Chaco have been paralleled by radiogenic strontium isotope studies designed to locate sources for wood recovered from Chaco Great Houses. More than 80% of the primary wooden beams in Great Houses were ponderosa pine (*Pinus ponderosa*), which grew in Chaco in the early 20^th^ century A.D., albeit only a few isolated trees [30]. However, many of the construction elements in Great Houses included Douglas fir (*Pseudotsuga menziesii*), spruce (*Picea spp.*) and even aspen (*Populus tremuloides*) species, which did not grow in Chaco during the late Holocene [31]–[33]. The ten largest ruins in Chaco would have required ca. 45,000 high elevation trees (2500 m and above today), based on species proportions recovered from excavations [34]. Archaeologists have argued that over time, Chaco builders would have depleted any locally available ponderosa, forcing them to seek this species in distant mountains, up to 80 km distant [34] [12] [28]. Spruce and fir in the region primarily occur in high-altitude sub-alpine montane and mesic woodlands in the Chuska, San Mateo, San Pedro, and La Plata Mountains while Ponderosa occupies the slopes of these ranges (Figure S4). These mountains ranges are at least 70+ kilometers away from Chaco Canyon Great Houses. However, spruce, fir, and ponderosa are sporadically available in mesic and riparian mixed conifer woodlands closer to the canyon (Figure S5) [41]. These trees typically grow on north-facing slopes near arroyos and other sources of periodic water. These woodlands occur along the paths to most nearby mountain ranges (Figure S6, S7, S8, S9). It is unlikely that these trees grew directly in Chaco Canyon based on macrobotanicals found in packrat middens in the canyon [33], though only two of these samples fall within a few centuries of the construction period.

Following a pioneering investigation which demonstrated that trees growing on different substrates in the San Juan Basin produced chemically distinct signatures [34], English, et al. (2001) analyzed radiogenic strontium isotopes from *Picea sp.* (spruce), *Abies sp.* (fir), *Pinus ponderosa* (ponderosa), water, and rocks at three areas where high elevation trees grow now. They compared these values to 52 archaeological specimens of spruce and fir from six Great Houses and concluded the archaeological wood came from the San Mateo Mountains (80+ km to the south) and the Chuska Mountains (75+ km to the west) [12]. Because these species correspond to modern elevations above 2500 m, the analysis did not include other possible geographic sources. Today, isolated stands of spruce, fir, and ponderosa exist in riparian and mesic mixed-conifer forests in the San Juan Basin [41]. Historically, isolated fir and ponderosa were both available in the canyon itself [27]. If conditions were previously cooler and wetter, these isolated stands could potentially have existed in larger quantities across the San Juan Basin [13] [14] and provided a more attractive source for timber than distant mountain ranges.

However, a subsequent attempt to use radiogenic strontium isotopes for sourcing ponderosa pine was unable to establish clear connections between potential source areas and archaeological specimens recovered from canyon Great Houses. Reynolds et al (2005) compared ^87^Sr/^86^Sr values for 62 living ponderosa from 19 geological source areas with 53 archaeological ponderosa from Pueblo Bonito, Chetro Ketl, and Pueblo del Arroyo (Figure 1). The results were plotted as means and the geological sample distribution compared visually to the archaeological distribution. Data were insufficient to allow clear identification of the particular origins of any of the Chaco timbers, although the authors suggested that the ponderosa pine data indicated a shift to more northern and western sources, specifically the La Plata or San Juan Mountains, ca. 150 km away. They also noted that they could not rule out the possibility that half the archaeological beams grew in Chaco itself, based on isotopic values from water samples in the canyon [15].

As geochemical data are variable due to geologic process or instrumental error, there must be some consideration of uncertainty in the original geochemical source data before attempting to make an argument for a specific geospatial source [42]. The present study uses both frequentist inference and Bayesian mixing models to assess the distinction between potential geochemical sources for the maize and trees found in Chaco Canyon by characterizing variation in both source samples and archaeological specimens as probability statements and distributions.

The sample of potential sources for spruce, fir and ponderosa was expanded beyond only high-altitude regions to include the Chaco Watershed and Aztec ruins based on riparian and mesic mixed conifer forest located in the area (Figures S4, S5, S6, S7, S8, S9). The sample of potential sources for maize includes the Chaco Watershed. This was excluded in earlier analysis as environmental conditions were not considered suitable for maize cultivation [7]–[10]. However, recent work indicates that maize cultivation was possible [16]–[17], as water control features and field houses are present in the canyon.

The large quantity of available radiogenic strontium data not only provides a way to get a more holistic view of the prehistoric economy of Chaco Canyon, but also a large data set to assess the utility of Bayesian inference into geochemical sourcing questions.

## Methods

Strontium data reported by English et al. (2001) and Reynolds et al. (2005) were used to define high-altitude geochemical sources for timber, while strontium data reported by Benson et al. (2003), Benson et al. (2009), and Benson et al. (2010) were used to define lower-elevation geochemical sources for maize cultivation in the immediate Chaco region and the broader San Juan Basin (Tables S9, S10, S11, S12) [12] [2] [15] [7]. Potential maize geochemical sources were defined using soil and synthetic water ^87^Sr/^86^Sr values (n = 154) and potential timber geochemical sources were defined using geologic and modern tree ^87^Sr/^86^Sr values (n = 223). Soil and water samples grouped by Benson et al. (2009) as either Chaco Canyon or the Upper Rio Chaco region were combined to form a single strontium isotope ratio set because the canyon is located in the hydrological center of the samples identified as the Upper Rio Chaco (Figure 2). This sample is referred to in this manuscript as the Chaco watershed. ^87^Sr/^86^Sr values for Chaco maize were grouped into temporal cultivation periods (pre-1140, post-1140, and historic). Values for archeological timbers were grouped by taxon (spruce, ponderosa, and fir). NBS SRM-987 values were reported for each study (English et al (2001) = 0.7102453+/−0.000012 [12]; Reynolds et al (2005) [15]  = 0.7102680+/−0.00004; Benson et al (2003) [2]  = 0.710276+/−0.000016; Benson et al (2009) [7]  = 0.71029+/−0.00002) and ^87^Sr/^86^Sr values were normalized to 0.71029 per Benson et al (2009) (Tables S9–S12).

Frequentist statistics can be used to determine whether or not ^87^Sr/^86^Sr measures of central tendency for potential sources, maize, or timber species are distinguishable. First, kernel density estimates and plots were created in order to visualize the probability distributions of ^87^Sr/^86^Sr values for sources, timber, and maize. Kernel density estimates were formed with the “stats” package in the R programming language (R 3.0.2) [43] [44] using a Gaussian smoothing kernel with a smoothing bandwidth equal to the standard deviation of each geochemical source and the Great House construction timber. These plots function similarly to histograms, but can be “smoothed” by assuming a normal distribution for each data point in order to depict a more curve-like probability density function for the variable of interest. For each geochemically defined source, timber taxon, and temporal maize cultivation group, 552 equally spaced points were generated with these smoothing parameters. The resulting kernel density estimate plots were used to produce individual ^87^Sr/^86^Sr probability curves for each geochemically defined source, each timber taxon, and each temporally defined maize cultivation groups. In addition to qualitative assessments of kernel density plots, simple Wilcoxon Mann-Whitney tests, which allow for comparing medians between data sets that are not normally distributed or have unequal variances, were run to determine if means were significantly different.

As a supplement to frequentist methods, mixing models can be used to assess possible source attributions [45]. Mixing models are most commonly used in ecology for assessing the contributions of different prey items to a predator's diet [46] [47], but they are well suited for geochemical source analysis as well. In the traditional approach, after measuring N isotopic elements, basic mixing models estimate the proportional contribution of N+1 sources to a mixture by solving a system of N+1 linear equations. A clear limitation to this method is that the number of sources considered in a model is capped by the number of isotopes measured [46] [48]. The use of Bayesian inference techniques allows a release from this restriction; theoretically any number of sources could be considered regardless of the number of isotopic elements [49] [50] [51]. A Bayesian approach also allows the incorporation of prior information into the model and more ways to investigate uncertainty and variability within potential sources. In this study, the R package SIAR (Stable Isotope Analysis in R) [49] [52] was used to build Bayesian mixing models to estimate the relative contribution of geochemical sources to the radiogenic strontium signatures in Chaco maize and timber. Using SIAR, each potential geochemical source was defined as a mean and standard deviation of measured ^87^Sr/^86^Sr from the soil, water, or modern trees in that locality. Archeological samples were defined using radiogenic ^87^Sr/^86^Sr values grouped by cultivation period (for maize: pre-1140, post-1140, and historic) or by taxonomic group (for timbers: spruce, ponderosa, and fir).

The Dirichlet distribution (a generalization of the more commonly used Beta distribution) was used as a prior for the proportional contribution of each source and no other information was included in the prior. Each source was treated as independent with an equal prior probability (12.5%), but the total proportions were required to sum to one [49]. Priors for all samples were kept as equal (12.5%) so that posterior probabilities primarily reflected the data following procedures used by Parnell et al. [49]. Isotopic data for each timber and maize sample were inputted, and 200,000 Markov Chain Monte Carlo simulations with 50,000 burn-ins were run to determine the proportionate contribution of each geochemical source to the maize and timbers of Chaco Canyon. Definitive source attributions should show a high proportionate contribution for one source over the others, while ambiguous source attributions should show an even spread of posterior probabilities.

One limitation of the mixing model software is that all data is assumed to follow a normal distribution [45] [49] [50]. To our knowledge, software does not yet exist to run Bayesian mixing models without this assumption. Shapiro-Wilkes tests for normality (Table S1) indicate this is a potential issue with the Chuska Mountains, San Pedro Mountains, and Aztec soils. To test the effects of these influences, a reduced model of the data were run for ponderosa with the Chuska Mountains corrected for normality (removing data points CHM-214b and CHM-214d) and for Pre-1140 A.D. maize (removing data point CHCU43684A). Removing these data limit the generalizability of these models, but do allow for a comparison between the posterior probabilities of data coerced to be normal to the full data set. A third robustness test included a sequential increase of prior probabilities by 3.5% from 12.5% to 93% for the Chuska Mountains with the posterior probability assessed relative to the prior probability.

## Results

### Maize Sources

**Figure 3:**
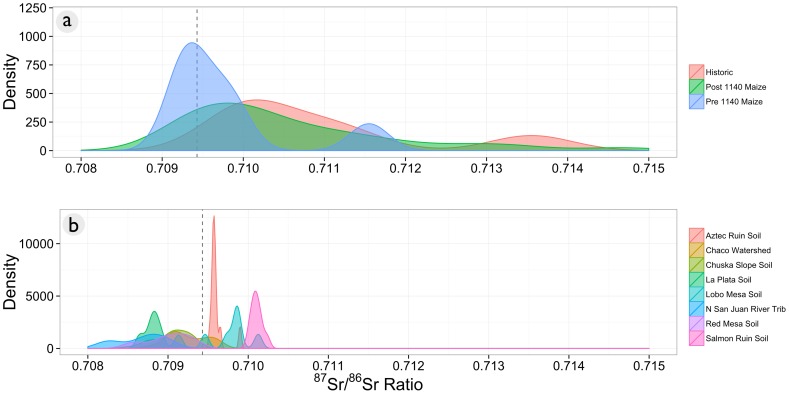
Kernel density distributions of historical, pre-1140 A.D., and post-1140 A.D. maize in Chaco Canyon (a) and soil and water ^87^Sr/^86^Sr sources (b). The dotted line represents the strontium isotope ratio for a single ponderosa tree stump found in the West Court of Pueblo Bonito (JPB-99). Maize from both time periods overlaps with strontium isotope ratios from the Chaco watershed, though maize dating after 1140 A.D. has more cobs with higher values than found within the canyon and in the selected source data set.

Our kernel density plots yielded a series of overlapping probability curves that reveal considerable uncertainty in source attribution for maize. ^87^Sr/^86^Sr values from Chaco Canyon maize radiocarbon-dated to Pueblo I and II periods (pre-1140 AD) largely overlap with the ^87^Sr/^86^Sr values of the Chaco watershed source, as well as many of the other defined sources (Figure 3a, 3b). Additionally, this subset of maize data has a bimodal distribution. A second, smaller peak appears outside the ^87^Sr/^86^Sr value of any known geochemical source within the study area. This indicates that there must be some yet-undefined geochemical source influencing this maize sample; it cannot be all sourced within the known geochemistry of the sampled areas.

Wilcoxon Mann-Whitney tests showed no statistical different in the ^87^Sr/^86^Sr values for maize predating 1140 A.D. and the Chaco watershed, Aztec soils, Chuska Slope soils, Lobo Mesa soils, or Salmon Ruin soil sources. Shapiro Wilkes tests for normality indicate that the Aztec soil, pre-1140 A.D., and post-1140 A.D. maize are non-normal to the 99% confidence level.

**Figure 4:**
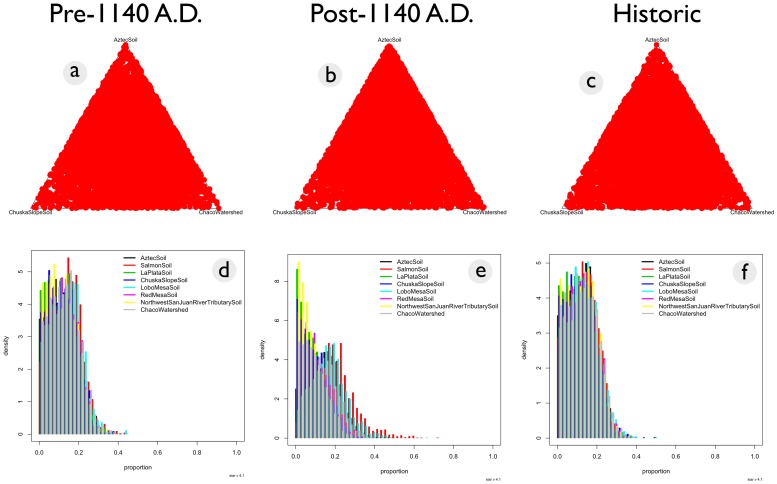
Ternary and proportion plots illustrating source mean posterior probabilities generated from Markov-Chain Monte Carlo simulations of strontium isotope data. There is no clear pattern for pre-1140 A.D. (a), post-1140 A.D. (b), and historic maize (c), indicating that no source attribution is more likely than others based on this model. Mean posterior probability proportions of geochemical source contributions for pre-1140 maize (d), post-1140 maize (e), and historic maize (f) are also displayed.

In the Bayesian mixing model results, Salmon Ruin had the highest mean source posterior probability for post-1140 A.D. maize (Figure 4a, Table 2), although it was not a large difference with respect to probability for the Aztec soil, Chaco watershed soil, or Lobo Mesa soil sources.

**Table 2:** Mean posterior probability proportions of geochemical source contributions to observed variation in Chaco maize.

	Pre 1140 A.D. Maize	Post 1140 A.D. Maize	Historic Maize
Chaco Watershed	0.127	0.145	0.128
Aztec Soil	0.131	0.114	0.135
Chuska Slope	0.120	0.098	0.119
Northwestern San Juan	0.110	0.076	0.121
Lobo Mesa	0.134	0.177	0.132
Red Mesa	0.119	0.094	0.122
La Plata	0.117	0.085	0.119
Salmon Ruin	0.143	0.211	0.125

For all maize samples, the strontium shows uniform mean posterior probabilities across all possible sources. This indicates a high degree of uncertainty when attempting to use strontium to source maize samples.

^87^Sr/^86^Sr values from maize grown in the Pueblo III period (post-1140 AD) partially overlapped with canyon drainage ^87^Sr/^86^Sr values, but some cobs had ratios outside those obtained from the Chaco watershed. These outlying ratios did not correspond to any other sources in this dataset (Figure 3a, 3b), suggesting an unknown or un-sampled source. It is certain that some post-1140 AD maize cobs were from the Navajo occupation of the canyon, which post-dates the acquisition of horses and consequently could easily have been transported from farming locales outside Chaco proper. This later maize had a small statistically significant difference with all potential sources except the Salmon Ruin area (Table 1). In the Bayesian mixing models, it had the highest probability of association with Salmon Ruin and Lobo Mesa soil sources, although again the differences are not great (Figure 3a, Table 2).

To determine the effects of non-normality on the conclusion for Pre-1140 A.D. maize, a sample (CHCU43684A) was removed from this data set and the model re-run without it. Mean posterior probabilities were not substantially different as a consequence of a violation of the normality assumption for this data set (Table S3). A second run of the model was performed on data rounded to the 4th decimal place, and neither median difference was significance (Table S4) or posterior probabilities (Table S5) were strongly affected by this treatment.

**Figure 5:**
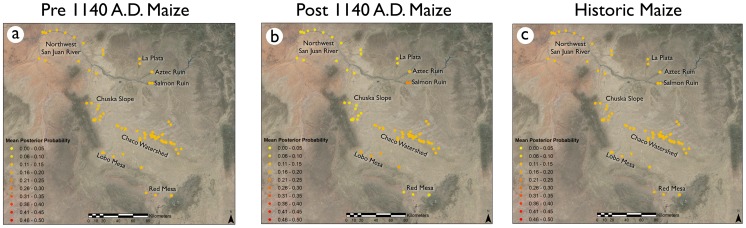
Map of mean posterior probabilities for source contribution in the San Juan Basin for pre-1140 A.D. maize (a), post-1140 A.D. maize (b), and historic maize (c).

Maize dated to the historic era (post 1492 A.D.) were similar to pre-1140 A.D. maize in that no single source was dominant (Figure 4c, Figure 5a–c, Table 2). Overall, the Monte Carlo simulations did not support any definitive geochemical sources associated with Chaco maize from any period (Table 2, Figure 4a–c, Figure 5a–c).

### Timber Sources

**Figure 6:**
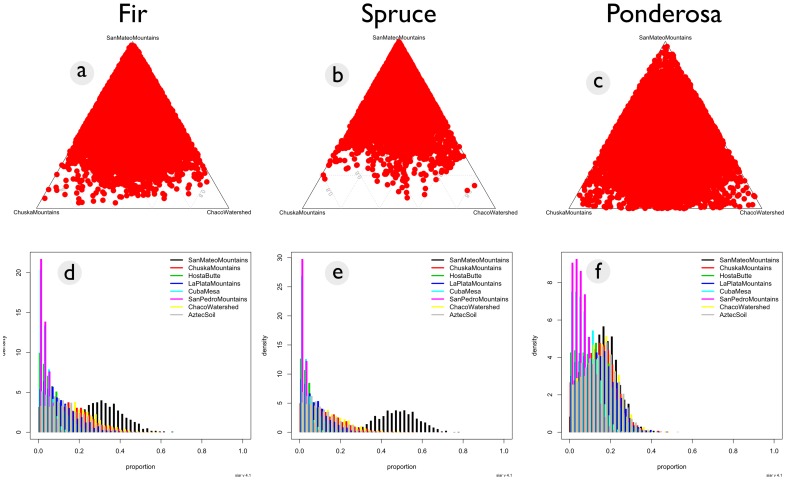
Ternary and proportion plots illustrating source posterior probabilities generated from Markov-Chain Monte Carlo simulations of strontium isotope data. Both Spruce and Fir (a & b) indicate San Mateo is a more likely source than either the Chuska Mountains or isolated stands near Chaco. There is no clear pattern for Ponderosa pine (c), indicating that no source attribution is more likely than others based on this model. Mean posterior probability proportions of geochemical source contributions for fir (d), spruce (e) and ponderosa (f) are also displayed.

**Figure 7:**
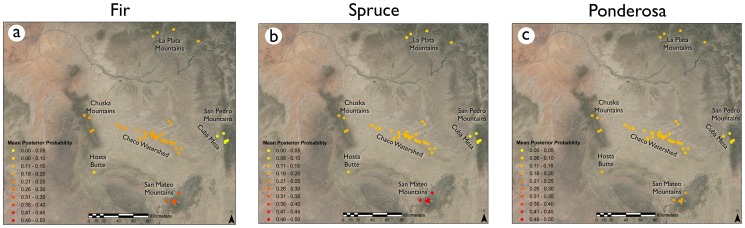
Map of mean posterior probabilities for source contribution in the San Juan Basin for fir (a), spruce (b), and ponderosa (c).

Wilcoxon Mann-Whitney tests indicated that there was no significant difference in medians for the Chaco watershed region and the Chuska Mountains at the 99% confidence level (Table 3), meaning these sources may not be sufficiently geochemically distinguishable. This complicates arguments for the Chuska Mountains as a major source for timber or maize [2]. The Bayesian mixing model indicated that some spruce and fir from Great Houses was likely from the San Mateo range (Figure 6a–f, Figure 7, Table 4), though some values fell within the range of the Chuska and Chaco watershed regions. It is important to note that the Bayesian model is not a confirmation that San Mateo is a source for construction timbers, only that it is the most likely given the available ^87^Sr/^86^Sr values. Although Chaco is a marginal location for ponderosa and fir today, and well outside the modern range of spruce, if trees that grow in Chaco Canyon mirror the strontium isotope values from the Chuska Mountains and, in part, the La Plata Mountains, it may be impossible to distinguish these two/three geochemical sources based on strontium isotope data alone. Therefore, the geochemical source attribution for timber data is also unclear.

**Table 3:** Comparison of medians across multiple potential timber sources.

	Chuska Mountains	San Mateo Mountains	Cuba Mesa	Hosta Butte	La Plata Mountains	San Pedro Mountains	Great Houses Ponderosa	Great Houses Spruce	Great Houses Fir
Chaco Watershed	−0.0002	0.0016**	−0.0049**	−0.0017**	−0.0006**	−0.0052**	−0.0003**	0.0007**	−0.0002
Chuska Mountains		0.0018**	−0.0046**	−0.0015**	−0.0003*	−0.0050**	−0.0001*	0.0009**	0.0001
San Mateo Mountains			−0.0064**	−0.0032**	−0.0021**	−0.0068**	−0.0019**	−0.0009**	−0.0017**
Cuba Mesa				0.0032*	0.0043**	−0.0003	0.0045**	0.0055**	0.0047**
Hosta Butte					0.0011	−0.0035**	0.0014*	0.0023**	0.0015**
La Plata Mountains						−0.0047**	0.0002	0.0012**	0.0004*
San Pedro Mountains							0.0049**	0.0059**	0.0051**
Great Houses Ponderosa								0.001**	0.0002**
Great Houses Spruce									−0.0008*

Significance codes: 0.001 ‘**’ 0.01 ‘*’.

Items in bold indicate that no significant difference between medians was found, potentially indicating that these sources cannot be quantitatively distinguished by strontium alone.

**Table 4:** Mean posterior probability proportions of geochemical source contributions to observed variation in Chaco timber.

	Spruce	Fir	Ponderosa
Chuska Mountains	0.121	0.153	0.145
Chaco Watershed	0.125	0.159	0.145
Aztec Soil	0.100	0.126	0.143
San Mateo Mountains	0.475	0.319	0.168
San Pedro Mountains	0.021	0.030	0.063
La Plata Mountains	0.078	0.108	0.135
Hosta Butte	0.056	0.072	0.124
Cuba Mesa	0.025	0.033	0.076

For most potential timber geochemical sources, mean posterior proportion probabilities show little difference from prior probabilities. This indicates a high degree of uncertainty when attempting to use strontium to source timber samples. Only the San Mateo source emerges as a higher probability source for spruce (0.475) and fir (0.319).

To determine the effects of non-normality on the conclusion for the Chuska source, two samples (CHM-214b and CHM-214d) were removed from this data set and the model re-run without it. Mean posterior probabilities were not substantially different as a consequence of a violation of the normality assumption for this data set (Table S6). A second run of the model was performed on data rounded to the 4th decimal place, and neither median was difference significance (Table S7) or posterior probabilities (Table S8) were strongly affected by this treatment. A sequential increase of prior probabilities increased posterior probabilities for the Chuska Mountains timber source for spruce, however in all cases the posterior was lower than the prior (Figure S10).

All code and analyses are available in the supporting information R code S1. All data is available in the supplementary material (Tables S9, S10, S11, S12). In addition, instructions are offered for modifying the code to use the same approach with new data.

## Discussion

**Figure 8:**
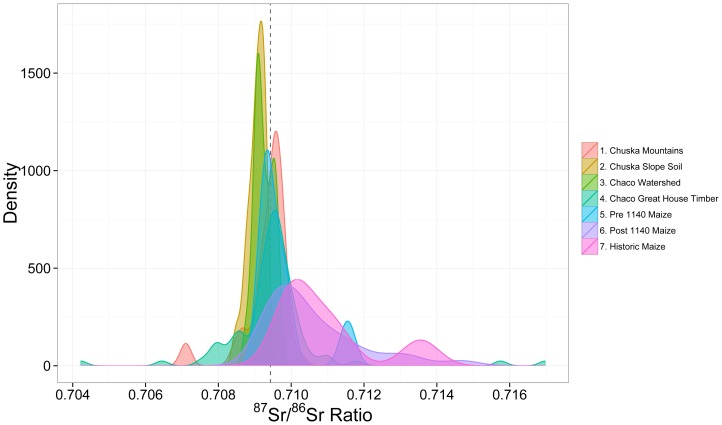
Correspondence of means between soils, maize, and trees in Chaco Canyon and the Chuska region. The red line represents the strontium isotope ratio for a Ponderosa tree stump found in the West Plaza of Pueblo Bonito (JPB-99). There is considerable overlap in strontium isotope ratios in the Chaco and Chuska regions.

Both qualitative interpretation using kernel density plots and quantitative source attributions using Bayesian mixing models indicate that geochemical sources for both maize and timber are not as distinguishable as indicated in the past, with the exception of the San Mateo Mountains. Data do not support the argument that maize was exclusively imported from outside Chaco Canyon, and do not support the Chuska Mountains as an unambiguous source for timber. There are no dominant or unambiguous geochemical, and thus geospatial, sources for Chaco maize or timber indicated in the radiogenic strontium isotope data. The overlapping kernel density plots for ^87^Sr/^86^Sr values of the Chaco watershed, maize cobs, and construction timbers from Great Houses, in addition to the Chuska Mountains and Chuska Slope soils, all share peaks near the ^87^Sr/^86^Sr value of the ponderosa stump (JPB-99) found in Pueblo Bonito (Figure 8). It is very likely that at least some of the geochemical sources of construction timber and maize are the same. ^87^Sr/^86^Sr values obtained from soil and water sources in the Chaco watershed are consistent with values from archaeological timbers from the major Great Houses (Figure 3a, 3b), but water samples from Werritos Rincon and South Gap (canyon tributaries) are also similar to the mean value for the Chuska sources, the lower mode for the La Plata source, and the Pueblo Bonito stump (JPB-99).

The results of robustness testing, which included removing data points to coerce the Chuska Mountains timber source and pre-1140 A.D. maize and rounding data to the 4th decimal place, did not substantially alter results or conclusions. This indicates that the Bayesian mixing model as employed in the SIAR package for R is robust to violations in the normality assumption, at least as far as the present study is concerned. These results mirror those of Parnell et al. 2010 [49] who found that SIAR was robust to violations of its core assumptions.

It is important to note how the Bayesian mixing models should, and should not, be interpreted. The posterior probability of the Bayesian mixing models should not be regarded as a hypothesis test for a given source, but rather as a statement of uncertainty regarding that source's contribution to the observed data in ecofacts from Chaco Canyon. Each source was assigned a prior probability of 12.5%. The posterior probability reflects the assessments of the likelihood of each source contributing to the variation observed in Chaco Canyon ecofacts in light of the strontium data from selected sources. That the posterior probability for the San Mateo Mountains, 47.5% (Table 4), is so much higher than the prior reflects the higher confidence of that range being a source for at least some of the spruce and fir. However, for ponderosa, the posterior probability, 14.5%, is not much higher than the prior; this indicates that strontium data does not present strong evidence for that range being a large proportional contributor to observed ^87^Sr/^86^Sr variation relative to other potential sources. This does not rule out San Mateo as a source for ponderosa, it only indicates that strontium data do not present a compelling case for this argument. This same interpretation extends to Chaco Canyon and the Chuska Mountains as sources for spruce, fir, or ponderosa; the posterior probabilities for proportional contribution are not substantially higher than the priors. Increasing the priors does not change this interpretation (Figure S10), as posterior probabilities remain lower than weighted priors. Likewise, for almost all maize sources for all time periods analyzed in the present manuscript, posterior probabilities for potential sources were not substantially higher than the priors used. The only exception to this is Salmon Ruins and possibly Lobo Mesa for the maize grown after 1140 A.D. but before the historic period. As noted earlier, posterior probabilities for source contribution to observed geochemical variability do not rule out potential sources, but they do address which sources are more or less likely to contribute to observed radiogenic strontium isotope ratio variation in ecofacts. It is difficult to make any clear arguments about geochemical sourcing for most maize or timber in Chaco Canyon in light of the currently available radiogenic strontium isotope data.

The clearest possible source attribution that emerged from the mixing model analysis is the San Mateo range for spruce and fir trees (Figure 6–7, Table 4). However, while the Bayesian approach indicated San Mateo was a likely source for some spruce and fir, Mann-Whitney tests showed significant differences between the medians of San Mateo ^87^Sr/^86^Sr values and the species means from construction timber in Chaco Canyon. These conflicting results highlight the limitations of frequentist methods when inferring geochemical sources. A geochemical source can neither be ruled out nor confirmed based solely on a shared mean/median with another geochemical data set, because an object can be collected from a limited portion of the source and thus have a significantly different ^87^Sr/^86^Sr value from its parent population. A comparison of means can indicate if a particular geochemical source is likely, but it cannot rule one out. However, a comparison of means can be highly useful in determining if geospatially distinct sources can be differentiated geochemically. For example, the Chuska range is distinct from the Chaco watershed as a geospatial source (Figure 1), but geochemically, they overlap almost completely (Figure 8, Table 3). The Bayesian mixing model approach illustrated in this manuscript offers a more probabilistic assessment for relative source contributions. Frequentist methods are appropriate when addressing if one geochemical source can be distinguished from another; Bayesian methods can be appropriate when assessing the probability of a given geochemical source explaining variation in a set of objects.

Maize data are particularly ambiguous; posterior probabilities do not substantially deviate from the priors used for source contribution, with the exception of Salmon Ruins for Post-1140 A.D. samples (Table 2). The central problem is that variation in maize ^87^Sr/^86^Sr values from all time periods overwhelms variation in geochemical sources (Figure 3). This phenomenon is interesting for two reasons. First it indicates that not all radiogenic strontium isotope variability has been documented in the San Juan Basin despite dedicated sampling and effort, a result identified by Benson [7]–[10]. Secondly, that historical maize largely matches the distribution of post-1140 A.D. maize and has a similarly large range as pre-1140 A.D. maize. As the ancestral pueblo were the primary occupants in the pre-historic period and the Navajo for the historic period [26], this raises interesting questions regarding long-distance transportation of maize. If, as argued in the past [7]–[10], maize was transported long distances to Chaco Canyon during the Great House period as based on ^87^Sr/^86^Sr values, why would historic maize follow the same pattern? Was historic maize imported from the same locations by a different ethnic group hundreds of years after Chaco's role as a ceremonial center had ended? Unfortunately, the strontium data are too ambiguous to facilitate a straightforward interpretation of provenance for existing maize samples.

The inconclusive results are not surprising because the San Juan Basin consists largely of sedimentary sandstones and shales that contribute through aeolian and alluvial processes to soil development [53]. Chaco Canyon is 32 km in length and includes an 11,000 sq. km watershed that funnels sediment into the canyon and its tributaries. The geological formations that contribute to the Chaco watershed (primarily the Mesa Verde and Meneffee formations) are widespread across the San Juan Basin (Figure 1). Consequently, it seems likely that canyon and basin soils would be relatively homogenized with respect to strontium and therefore it is difficult to differentiate from any particular source in the larger region [54] [55]. Based on available radiogenic strontium isotopes samples, Chaco Canyon and its tributaries are equally likely to represent source areas for ponderosa as far more distant parts of the San Juan Basin. Taken in isolation, radiogenic strontium isotope ratios from the Chuska Mountains region is consistent with data from Chaco Canyon, but the same statement can be made for a much broader area across the entire Colorado plateau that, in wetter conditions, could have supported more forests in and near riparian and mesic mixed conifer forests present in the basin.

The results of quantitative analysis indicate considerable ambiguity regarding geochemical sources for Chaco canyon maize and tree lumber. While some spruce and fir trees can likely be sourced to the San Mateo range, there is no clear evidence for a primary ponderosa geochemical source. A few ponderosa stands may be sourced to the San Pedro range due to a high ^87^Sr/^86^Sr value [15], but it is unlikely that the San Pedro range was a major source for timber. Like ponderosa, maize from Chaco Canyon also does not have any clear geochemical sources (Figure 4a, Figure 5a–c). The data presented here do not contradict arguments that some construction elements may have originated from the San Mateo Mountains [12] [15], but there is no geochemical evidence that the Chuska Mountains were a major source for construction timbers. Similarly, the Chuska slope does not appear to be any more likely as a source for maize compared to more local alternatives (Figure 4a–c, Figure 5a, Table 2). The range of ^87^Sr/^86^Sr values for the Chuska Mountains and Chuska slope soils overlaps considerably with values of soils from the Chaco watershed (Figure 8), and there is not a significant difference between their means (Table 3). As such, isolated ponderosa stands near Chaco Canyon and a dense ponderosa forest in the Chuska Mountains will have the same strontium isotope ratio. Therefore, ^87^Sr/^86^Sr values cannot be used to distinguish between the Chuska and Chaco regions. For maize, Chuska slope soils and the Chaco watershed can be differentiated generally, but the majority of pre-1140 A.D. maize falls within the overlapping ^87^Sr/^86^Sr values that characterize both potential sources (Figure 3a,b).

Recent modeling of potential agricultural productivity in Chaco Canyon [16] [17] indicates that there was more arable land than recognized in previous studies of Bonito Phase food production. Without a clear indication from geochemical analyses that extramural sources of maize or ponderosa pine were important during the Bonito Phase, the case for long-distance transport of food and wood has not been demonstrated. Therefore there is no unambiguous evidence that the economic foundation of Chaco society was predicated on such transactions. Some high elevation wood was brought to the canyon, at least in part including the San Mateo Mountains, but it is unknown how these trees were procured or whether they were economically significant. For maize in particular, an argument for long-distance transport is tenuous due to the close correspondence of ^87^Sr/^86^Sr values of historical maize and ^87^Sr/^86^Sr values of post-1140 A.D. maize (Figure 3a), as well as the overlap in ^87^Sr/^86^Sr values from maize from all periods have with Chaco watershed ^87^Sr/^86^Sr values (Figure 3b).

The concept of a “source” in geochemistry studies is complex and varies considerably in spatial scale, from individual specimens or samples to geographic regions, and therefore sampling designs that match data to questions are critical to the success of any sourcing enterprise [56]. In using chemical data for sourcing, an important distinction must be drawn between geochemical sources and geospatial sources [42]. Potential sources for Bonito Phase maize may lie outside the canyon and it is possible that ponderosa pine was transported long distances to Chaco. Recent analysis suggests that the northern portion of the Chaco watershed may be a maize source; this would be supported in part by the present analysis. However, while some chert artifacts do originate for the Chuska Mountains region [57], the radiogenic strontium isotope evidence does not support this region as a likely source for either maize or construction timbers. San Mateo is better supported as a support by this analysis; obsidian has been demonstrated to have come, in part, from Mt. Taylor in the San Mateo Range [58]. However, the connection between timber acquisition and lithic procurement is ambiguous. As Chaco Canyon was a source of long-distance trade, it is difficult to weight the influences of one type of artifact upon another. The present re-evaluation of radiogenic strontium isotope data does not rule out some long-distance transport of high elevation tree species, though it best supports models in which these come from the south, not the west or north. The case for long-distance acquisition in maize and ponderosa pine is ambiguous and additional research is needed to determine possible relationships between Great Houses the procurement of these costly resources.

## Supporting Information

Figure S1 **Geological Labels.**
(TIF)

Figure S2 **Sediment Age of Deposition.**
(TIF)

Figure S3 **Sediment Type.**
(TIF)

Figure S4 **San Juan Basin Vegetation Cover.**
(TIF)

Figure S5 **Chaco Area Vegetation Cover.**
(TIF)

Figure S6 **Chaco/Chuska Mountains Vegetation Cover.**
(TIF)

Figure S7 **Chaco/San Mateo Mountains Vegetation Cover.**
(TIF)

Figure S8 **Chaco/San Pedro Mountains Vegetation Cover.**
(TIF)

Figure S9 **Chaco/La Plata Mountains Vegetation Cover.**
(TIF)

Figure S10 **Difference between Posterior and Prior probabilities for sequentially weighted priors for the Chuska Mountains as a potential source of spruce.**
(TIF)

Table S1 Shapiro-Wilkes Test for normality of sources.
(DOC)

Table S2 Shapiro-Wilkes Test for tested data.
(DOC)

Table S3 Posterior probabilities of maize data coerced to normality through removal of data points (Pre-1140 A.D. Maize, CHCU43684A) and full model results.
(DOC)

Table S4 Comparison of medians across maize by provenience (columns) and potential sources (rows). Items in bold indicate that no significant difference between medians was found. Original ^87^Sr/^86^Sr data were rounded to the 4th decimal place.
(DOC)

Table S5 Proportion of the 200,000 run simulations in which each maize sample (columns) fell within the mean +/− one standard deviation of each source (rows). For all maize samples, the strontium shows uniform proportions across all possible sources. This indicates a high degree of uncertainty when attempting to use strontium to source maize samples. Original ^87^Sr/^86^Sr data were rounded to the 4th decimal place.
(DOC)

Table S6 Posterior probabilities of source data coerced to normality through removal of data points (Chuska Mountains, CHM-214b and CHM-214d) and full model results.
(DOC)

Table S7 Comparison of medians across multiple potential timber sources. Items in bold indicate that no significant difference between medians was found, potentially indicating that these sources cannot be quantitatively distinguished by strontium alone. Original ^87^Sr/^86^Sr data were rounded to the 4th decimal place.
(DOC)

Table S8 Proportion of the 200,000 run simulations in which each timber sample (columns) fell within the mean +/− one standard deviation of each source (rows). For all timber samples, the strontium shows uniform proportions across all possible sources. This indicates a high degree of uncertainty when attempting to use strontium to source timber samples. Only the San Mateo source emerges as a higher probability source for spruce (0.469) and fir (0.320). Original ^87^Sr/^86^Sr data were rounded to the 4th decimal place.
(DOC)

Table S9 Maize Source Strontium Isotope Data.
(DOC)

Table S10 Tree Source Strontium Isotope Data.
(DOC)

Table S11 Maize Strontium Isotope Data.
(DOC)

Table S12 Chaco Timber Strontium Isotope Data.
(DOC)

R code S1 **Supplementary computer code.**
(TXT)
