# Correction: Strontium Isotopes and the Reconstruction of the Chaco Regional System: Evaluating Uncertainty with Bayesian Mixing Models

**DOI:** 10.1371/journal.pone.0105849

**Published:** 2014-08-11

**Authors:** The PLOS ONE Staff

The corresponding author’s email is incorrect. It should be b.lee.drake@gmail.com.

There is an error in the second sentence of the abstract, which should indicate that the Chuska Mountains are to the west of the Chaco Canyon. The correct sentence is: In Chaco Canyon, an Ancestral Pueblo regional center in New Mexico, previous studies using these methods have suggested that significant portion of maize and wood originate in the Chuska Mountains region, 75 km to the West.
