# Supplementary figures and images for: Strontium Isotopes and the Reconstruction of the Chaco Regional System: Evaluating Uncertainty with Bayesian Mixing Models
Source: PLoS One. 2014 May 22;9(5):e95580. doi: 10.1371/journal.pone.0095580 (PMC4031078; doi:10.1371/journal.pone.0095580)

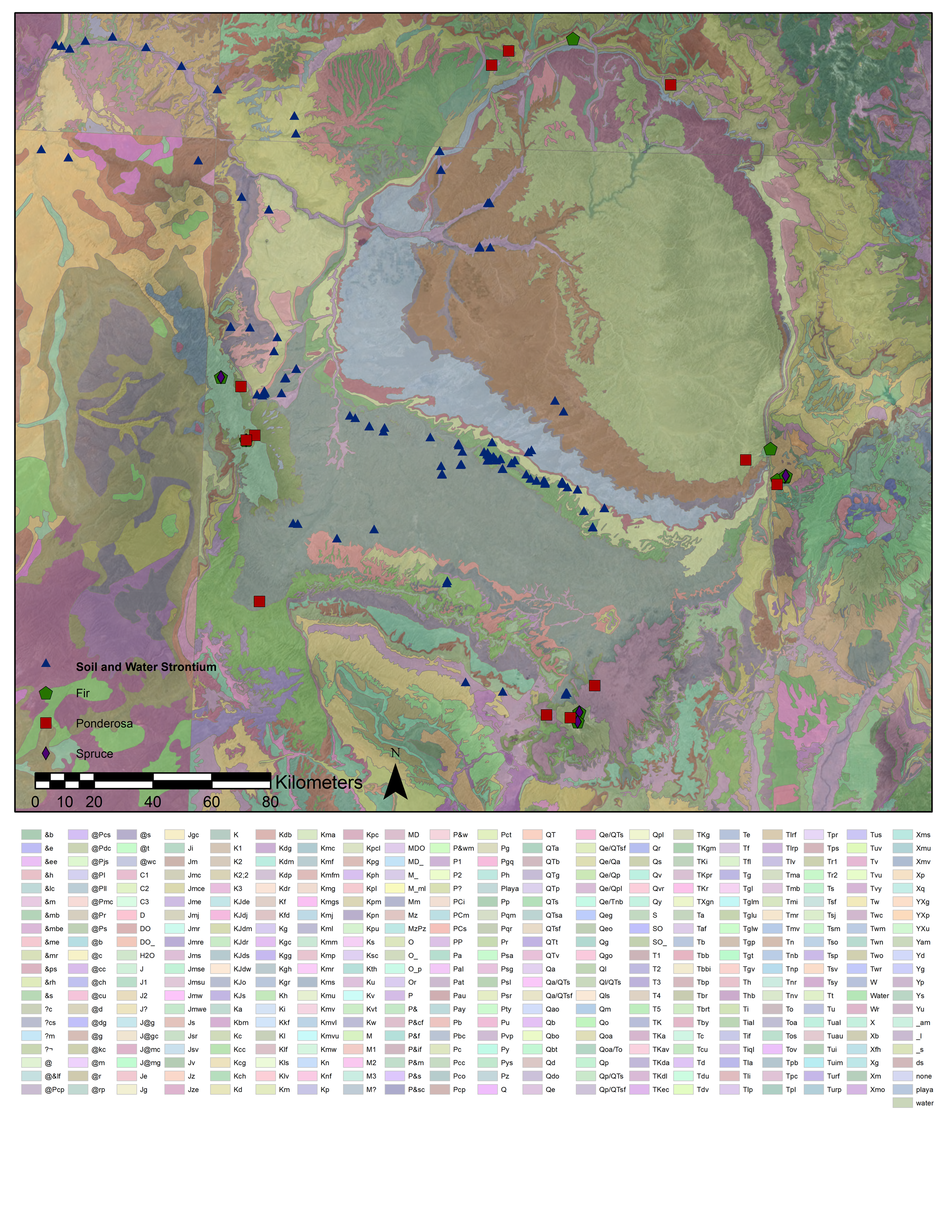

Supplement: Figure S1 — Geological Labels. (TIF) [file pone.0095580.s001.tif]

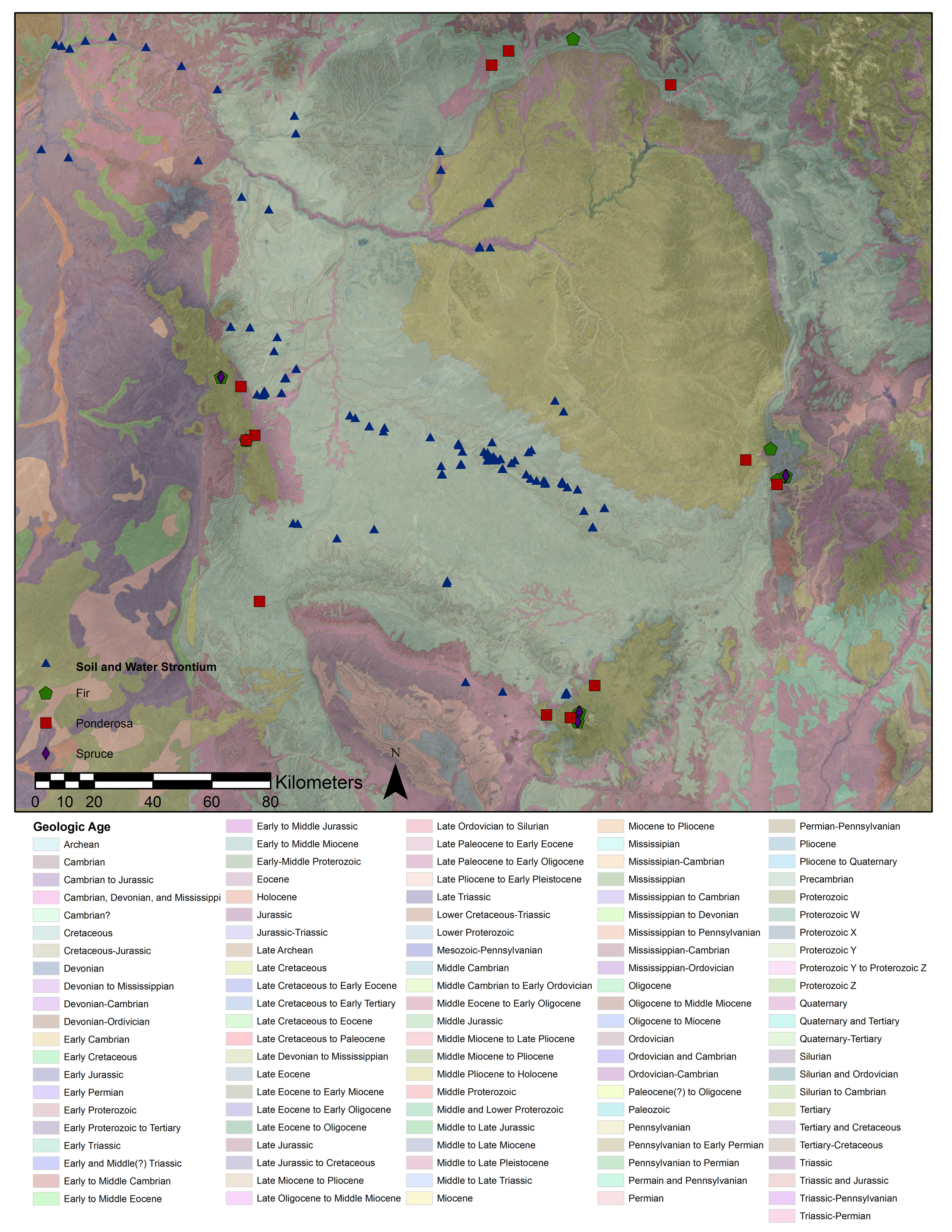

Supplement: Figure S2 — Sediment Age of Deposition. (TIF) [file pone.0095580.s002.tif]

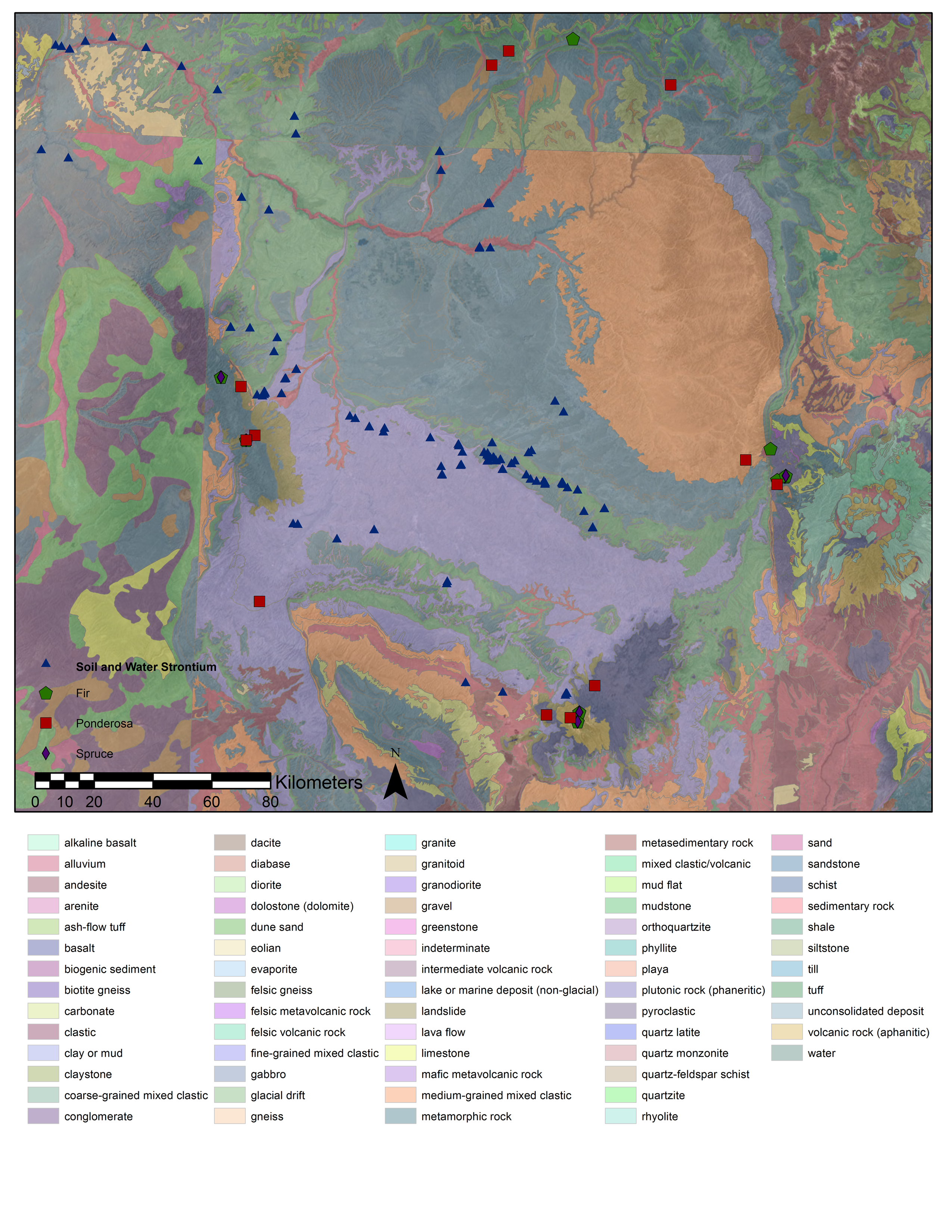

Supplement: Figure S3 — Sediment Type. (TIF) [file pone.0095580.s003.tif]

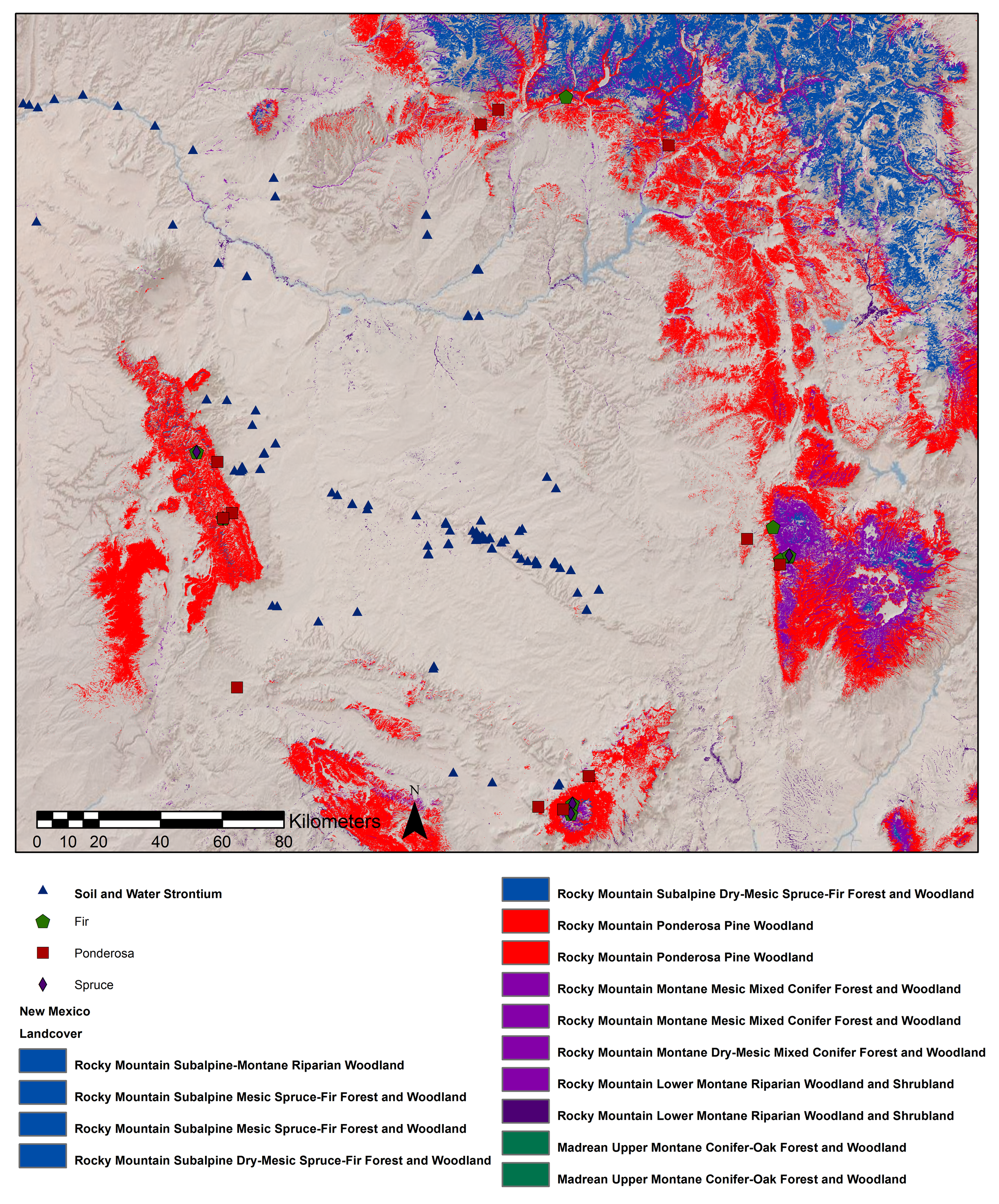

Supplement: Figure S4 — San Juan Basin Vegetation Cover. (TIF) [file pone.0095580.s004.tif]

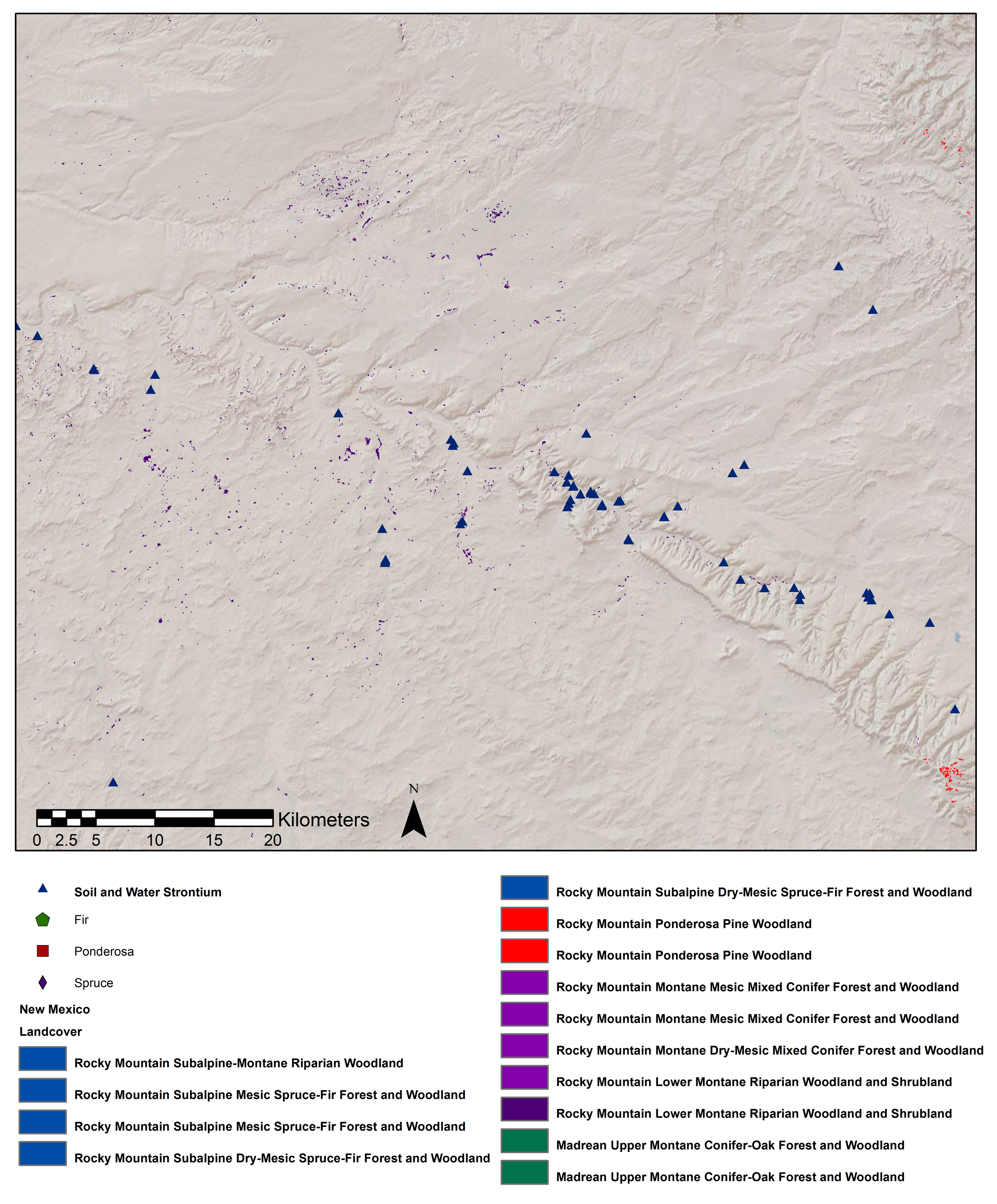

Supplement: Figure S5 — Chaco Area Vegetation Cover. (TIF) [file pone.0095580.s005.tif]

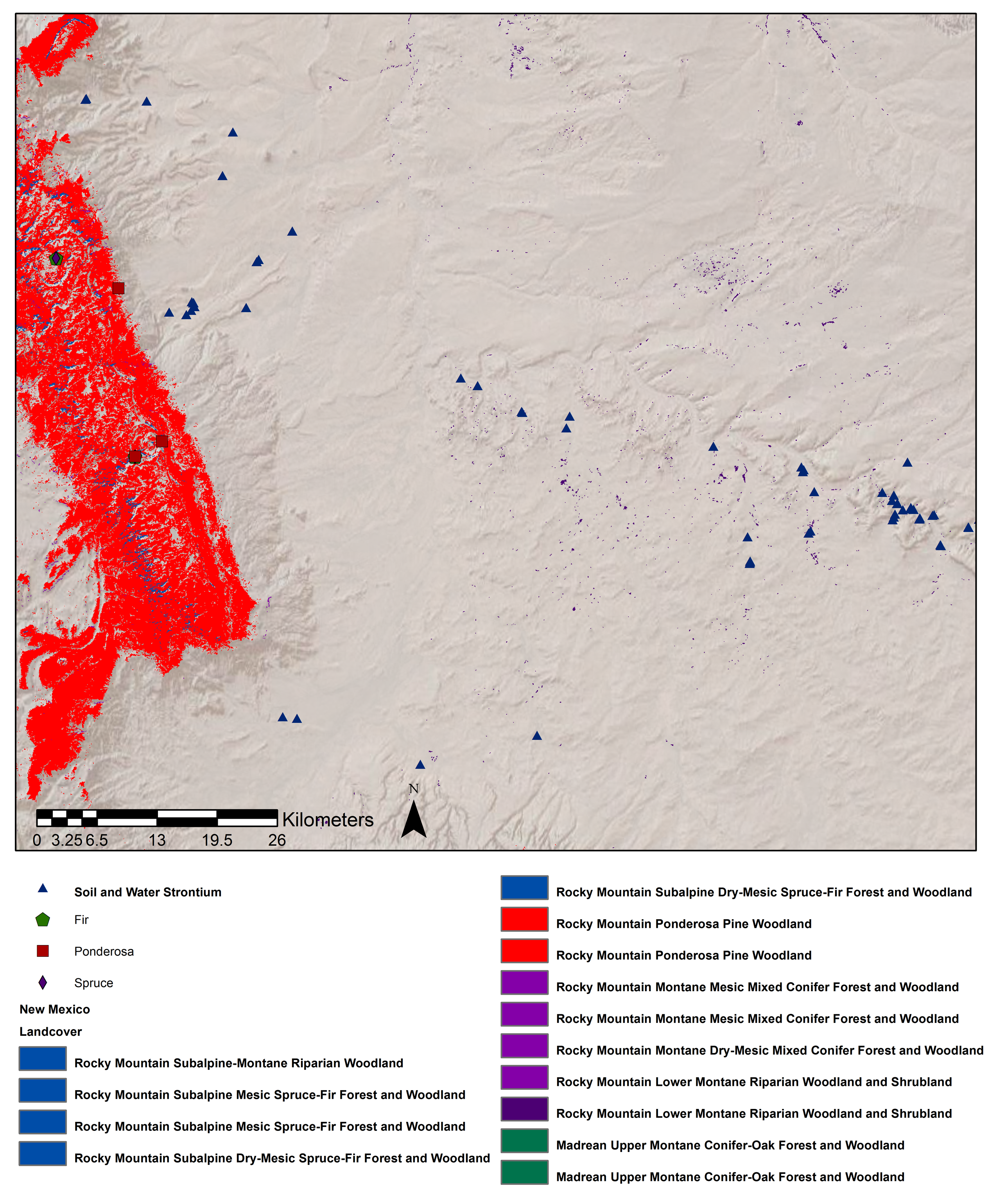

Supplement: Figure S6 — Chaco/Chuska Mountains Vegetation Cover. (TIF) [file pone.0095580.s006.tif]

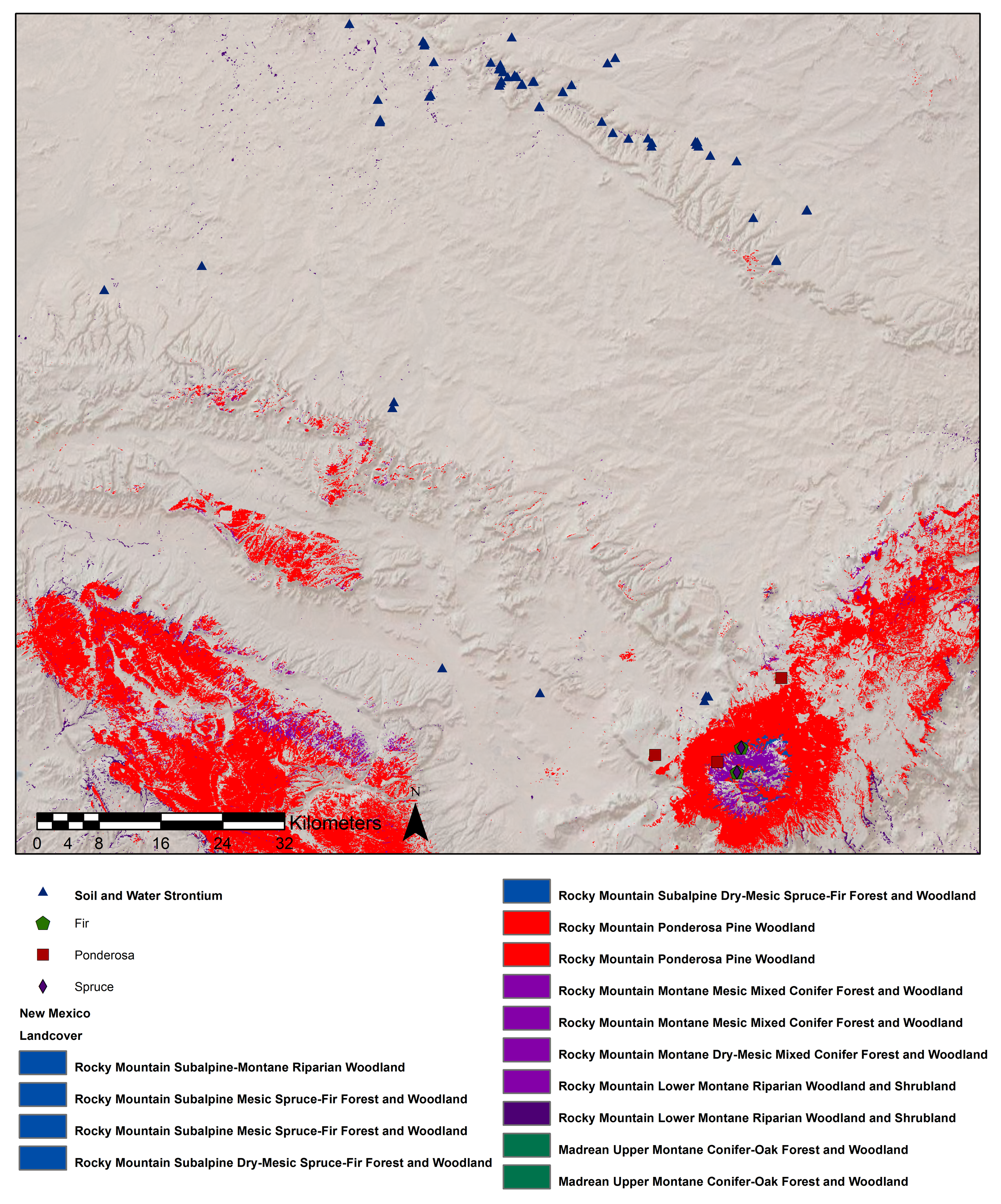

Supplement: Figure S7 — Chaco/San Mateo Mountains Vegetation Cover. (TIF) [file pone.0095580.s007.tif]

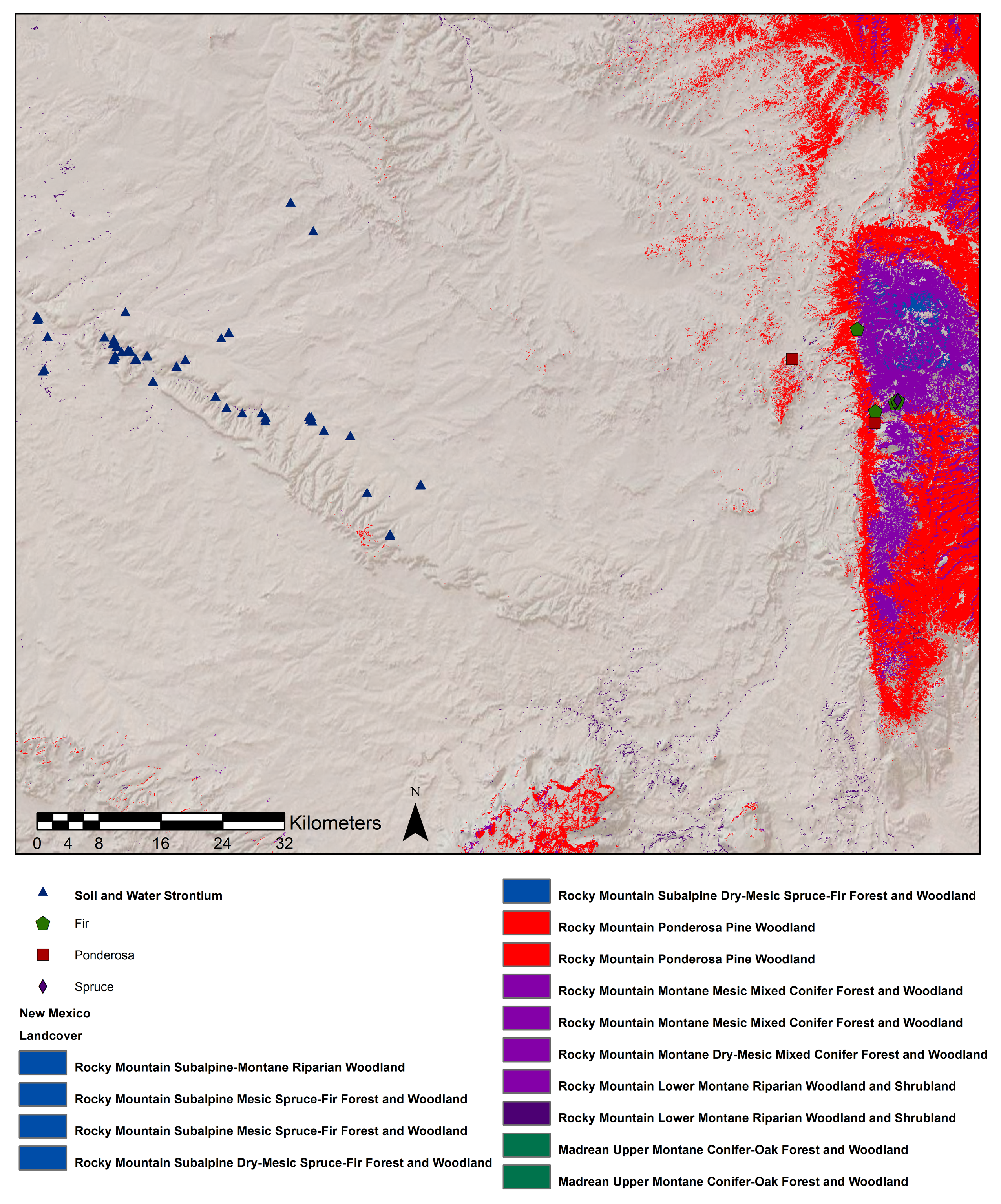

Supplement: Figure S8 — Chaco/San Pedro Mountains Vegetation Cover. (TIF) [file pone.0095580.s008.tif]

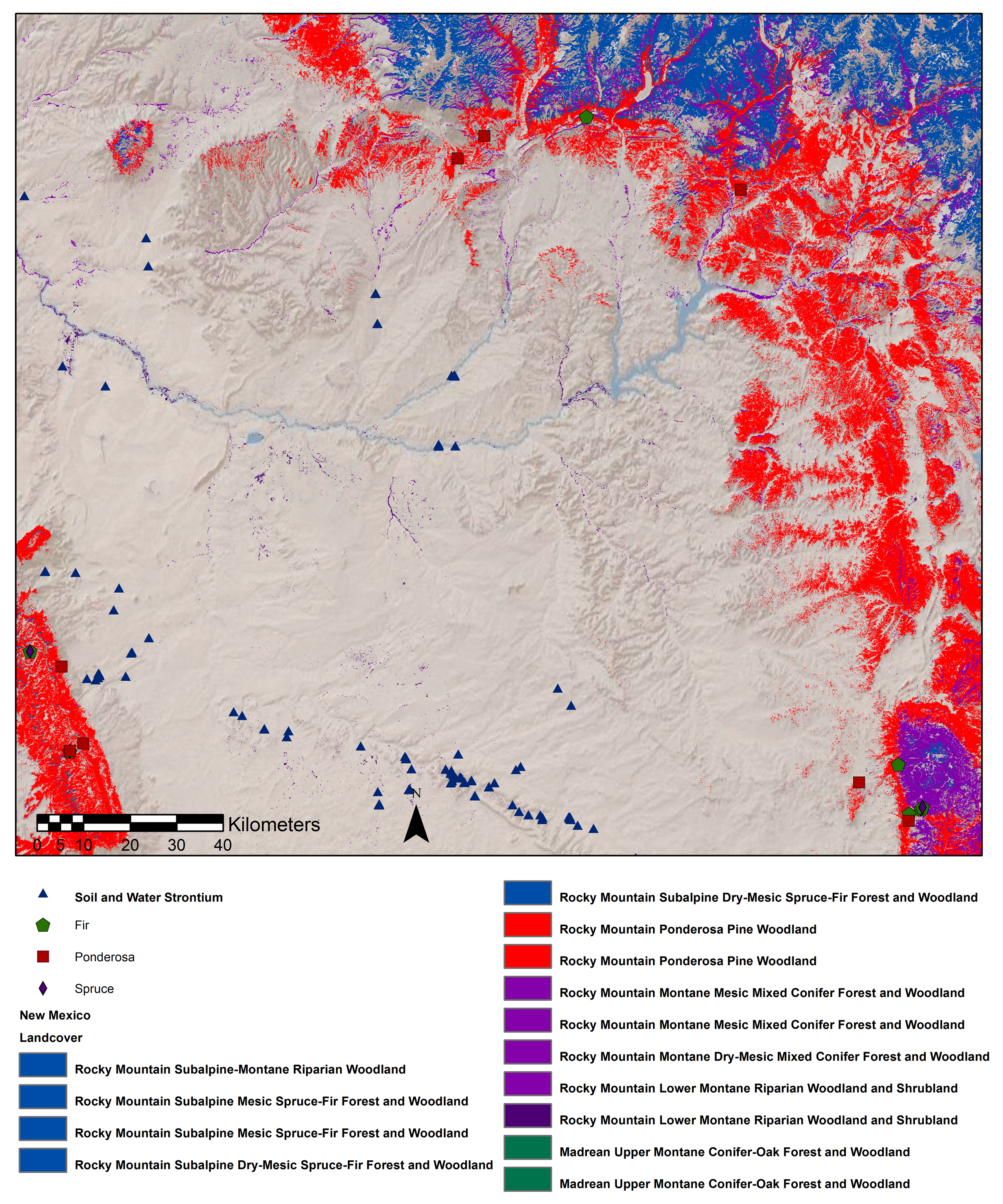

Supplement: Figure S9 — Chaco/La Plata Mountains Vegetation Cover. (TIF) [file pone.0095580.s009.tif]

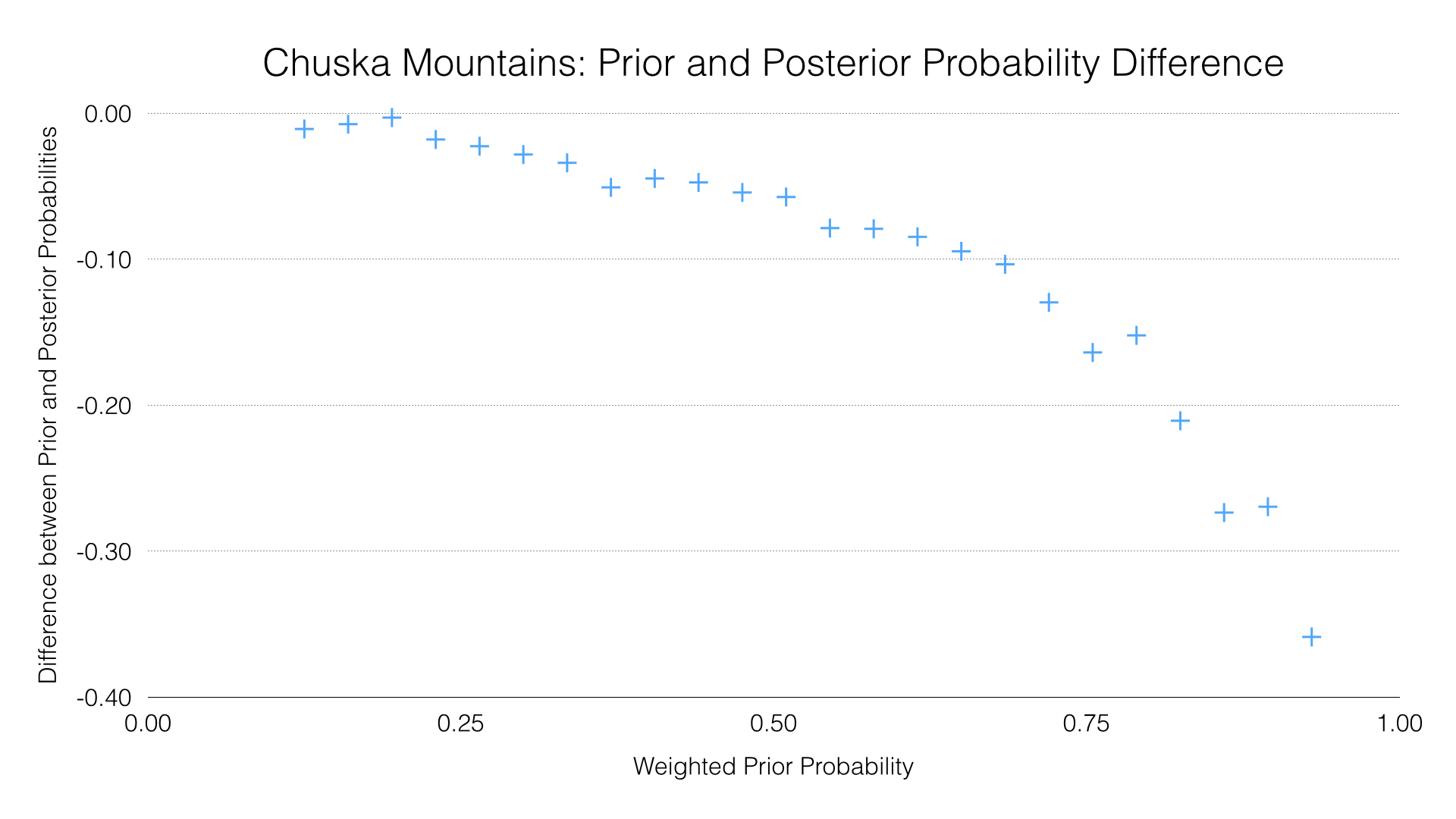

Supplement: Figure S10 — Difference between Posterior and Prior probabilities for sequentially weighted priors for the Chuska Mountains as a potential source of spruce. (TIF) [file pone.0095580.s010.tif]
