# Supplementary material for: Strontium Isotopes and the Reconstruction of the Chaco Regional System: Evaluating Uncertainty with Bayesian Mixing Models
Source: PLoS One. 2014 May 22;9(5):e95580. doi: 10.1371/journal.pone.0095580 (PMC4031078; doi:10.1371/journal.pone.0095580)
Supplement: Table S1 — Shapiro-Wilkes Test for normality of sources. (DOC) [file pone.0095580.s011.doc]

| Tree Sources | p-value | Maize Sources | p-value |
| --- | --- | --- | --- |
| San Mateo | 0.01473 | Aztec | **0.003048** |
| Chuska | **4.315E-07** | Salmon | 0.9711 |
| Hosta Butte | 0.4928 | La Plata | 0.6996 |
| La Plata | 0.1911 | Chuska Slope | 0.1457 |
| Cuba Mesa | 0.7079 | Lobo Mesa | 0.5382 |
| San Pedro | **0.001521** | Red Mesa | 0.8114 |
| Chaco | 0.07924 | Chaco | 0.07924 |
| Aztec | **0.003048** | San Juan | 0.05757 |
