# Supplementary material for: Strontium Isotopes and the Reconstruction of the Chaco Regional System: Evaluating Uncertainty with Bayesian Mixing Models
Source: PLoS One. 2014 May 22;9(5):e95580. doi: 10.1371/journal.pone.0095580 (PMC4031078; doi:10.1371/journal.pone.0095580)
Supplement: Table S2 — Shapiro-Wilkes Test for tested data. (DOC) [file pone.0095580.s012.doc]

| Tree | p-value | Maize | p-value |
| --- | --- | --- | --- |
| Spruce | 0.5169 | Pre-1140 A.D. | **0.005966** |
| Fir | **2.237E-07** | Post-1140 A.D. | **0.0001944** |
| Ponderosa | **3.259E-11** | Historic | 0.07218 |
