# Supplementary material for: Strontium Isotopes and the Reconstruction of the Chaco Regional System: Evaluating Uncertainty with Bayesian Mixing Models
Source: PLoS One. 2014 May 22;9(5):e95580. doi: 10.1371/journal.pone.0095580 (PMC4031078; doi:10.1371/journal.pone.0095580)
Supplement: Table S3 — Posterior probabilities of maize data coerced to normality through removal of data points (Pre-1140 A.D. Maize, CHCU43684A) and full model results. (DOC) [file pone.0095580.s013.doc]

| Pre-1140 AD | Original Data Set Mean Posterior Probability | Reduced Data Set Mean Posterior Probability |
| --- | --- | --- |
|  | *Does not conform to normal distribution* | *Does conform to normal distribution* |
| Chaco Watershed | 0.127 | 0.121 |
| Aztec Soil | 0.131 | 0.137 |
| Chuska Slope | 0.120 | 0.117 |
| Northwestern San Juan | 0.110 | 0.103 |
| Lobo Mesa | 0.134 | 0.146 |
| Red Mesa | 0.119 | 0.113 |
| La Plata | 0.117 | 0.109 |
| Salmon Ruin | 0.143 | 0.154 |
