# Supplementary material for: Strontium Isotopes and the Reconstruction of the Chaco Regional System: Evaluating Uncertainty with Bayesian Mixing Models
Source: PLoS One. 2014 May 22;9(5):e95580. doi: 10.1371/journal.pone.0095580 (PMC4031078; doi:10.1371/journal.pone.0095580)
Supplement: Table S4 — Comparison of medians across maize by provenience (columns) and potential sources (rows). Items in bold indicate that no significant difference between medians was found. Original 87Sr/86Sr data were rounded to the 4th decimal place. (DOC) [file pone.0095580.s014.doc]

|  | Aztec Soil | Chuska Slope | Lobo Mesa | Northwestern San Juan River | Red Mesa | La Plata | Salmon Ruin | Pre 1140 Maize | Post 1140 Maize | Historic Maize |
| --- | --- | --- | --- | --- | --- | --- | --- | --- | --- | --- |
| Chaco Watershed | -0.0003 | 0.0002* | -0.0006** | 0.0006** | 0.0003 | -0.0005** | -0.0008** | -0.0002 | -0.0008** | -0.0012** |
| Aztec Soil |  | 0.0005** | -0.0003 | 0.0009** | 0.0006* | 0.0008* | -0.0005* | 0.0001 | -0.0005 | -0.0009* |
| Chuska Slope |  |  | -0.0008** | 0.0004** | 0.0001 | 0.0003 | -0.001** | -0.0004** | -0.001** | -0.0014** |
| Lobo Mesa |  |  |  | 0.0012** | 0.0009* | 0.0011* | -0.0002 | 0.0004 | -0.0002 | -0.0006 |
| Northwestern San Juan River |  |  |  |  | -0.0003 | -0.0001 | -0.0014 | -0.0008** | -0.0014** | -0.0018** |
| Red Mesa |  |  |  |  |  | 0.0002 | -0.0011 | -0.0005 | -0.0011** | -0.0015* |
| La Plata |  |  |  |  |  |  | -0.0013* | -0.0007* | -0.0013** | -0.0017* |
| Salmon Ruin |  |  |  |  |  |  |  | 0.0006 | 0 | -0.0004 |
| Pre 1140 Maize |  |  |  |  |  |  |  |  | -0.0006 | -0.0001 |
| Post 1140 Maize |  |  |  |  |  |  |  |  |  | -0.0004 |
|  | Significance codes: 0.001 ‘**’ 0.01 ‘*’ | | | | | | | | | |
