# Supplementary material for: Strontium Isotopes and the Reconstruction of the Chaco Regional System: Evaluating Uncertainty with Bayesian Mixing Models
Source: PLoS One. 2014 May 22;9(5):e95580. doi: 10.1371/journal.pone.0095580 (PMC4031078; doi:10.1371/journal.pone.0095580)
Supplement: Table S5 — Proportion of the 200,000 run simulations in which each maize sample (columns) fell within the mean +/− one standard deviation of each source (rows). For all maize samples, the strontium shows uniform proportions across all possible sources. This indicates a high degree of uncertainty when attempting to use strontium to source maize samples. Original 87Sr/86Sr data were rounded to the 4th decimal place. (DOC) [file pone.0095580.s015.doc]

|  | Pre 1140 Maize | Post 1140 Maize | Historic |
| --- | --- | --- | --- |
| Chaco Watershed | 0.123 | 0.116 | 0.123 |
| Aztec Soil | 0.131 | 0.147 | 0.127 |
| Chuska Slope | 0.119 | 0.097 | 0.121 |
| Northwestern San Juan | 0.109 | 0.079 | 0.118 |
| Lobo Mesa | 0.140 | 0.172 | 0.133 |
| Red Mesa | 0.120 | 0.097 | 0.125 |
| La Plata | 0.116 | 0.085 | 0.119 |
| Salmon Ruin | 0.142 | 0.208 | 0.134 |
