# Supplementary material for: Strontium Isotopes and the Reconstruction of the Chaco Regional System: Evaluating Uncertainty with Bayesian Mixing Models
Source: PLoS One. 2014 May 22;9(5):e95580. doi: 10.1371/journal.pone.0095580 (PMC4031078; doi:10.1371/journal.pone.0095580)
Supplement: Table S6 — Posterior probabilities of source data coerced to normality through removal of data points (Chuska Mountains, CHM-214b and CHM-214d) and full model results. (DOC) [file pone.0095580.s016.doc]

| Ponderosa Pine | Original Data Set Mean Posterior Probability | Reduced Data Set Mean Posterior Probability |
| --- | --- | --- |
|  | *Does not conform to normal distribution* | *Does conform to normal distribution* |
| Chuska Mountains | 0.145 | 0.144 |
| Chaco Watershed | 0.145 | 0.146 |
| Aztec Soil | 0.143 | 0.141 |
| San Mateo Mountains | 0.168 | 0.171 |
| San Pedro Mountains | 0.063 | 0.063 |
| La Plata Mountains | 0.135 | 0.137 |
| Hosta Butte | 0.124 | 0.123 |
| Cuba Mesa | 0.076 | 0.074 |
