# Supplementary material for: Strontium Isotopes and the Reconstruction of the Chaco Regional System: Evaluating Uncertainty with Bayesian Mixing Models
Source: PLoS One. 2014 May 22;9(5):e95580. doi: 10.1371/journal.pone.0095580 (PMC4031078; doi:10.1371/journal.pone.0095580)
Supplement: Table S7 — Comparison of medians across multiple potential timber sources. Items in bold indicate that no significant difference between medians was found, potentially indicating that these sources cannot be quantitatively distinguished by strontium alone. Original 87Sr/86Sr data were rounded to the 4th decimal place. (DOC) [file pone.0095580.s017.doc]

|  | Chuska Mountains | San Mateo Mountains | Cuba Mesa | Hosta Butte | La Plata Mountains | San Pedro Mountains | Great Houses Ponderosa | Great Houses Spruce | Great Houses Fir |
| --- | --- | --- | --- | --- | --- | --- | --- | --- | --- |
| Chaco Watershed | -0.0004* | 0.0016** | -0.0048** | -0.0017** | -0.0005** | -0.0052** | -0.0003** | 0.0007** | **-0.0002** |
| Chuska Mountains |  | 0.0018** | -0.0046** | -0.0015** | -0.0003* | -0.0050** | -0.0001* | 0.0009** | **-0.0001** |
| San Mateo Mountains |  |  | -0.0064** | -0.0033** | -0.0021** | -0.0068** | -0.0019** | -0.0001** | -0.0018** |
| Cuba Mesa |  |  |  | **0.0031** | 0.0043** | **-0.0004** | 0.0045** | 0.0055* | 0.0047** |
| Hosta Butte |  |  |  |  | **0.0012** | -0.0035** | 0.0014* | 0.0024* | 0.0016** |
| La Plata Mountains |  |  |  |  |  | -0.0047** | **0.0002** | 0.0012** | 0.0004* |
| San Pedro Mountains |  |  |  |  |  |  | 0.0050** | 0.0059** | 0.0051** |
| Great Houses Ponderosa |  |  |  |  |  |  |  | 0.0010** | 0.0002* |
| Great Houses Spruce |  |  |  |  |  |  |  |  | -0.0008* |
|  | Significance Codes: 0.001 ‘**’ 0.01 ‘*’ | | | | | | | | |
