# Supplementary material for: Strontium Isotopes and the Reconstruction of the Chaco Regional System: Evaluating Uncertainty with Bayesian Mixing Models
Source: PLoS One. 2014 May 22;9(5):e95580. doi: 10.1371/journal.pone.0095580 (PMC4031078; doi:10.1371/journal.pone.0095580)
Supplement: Table S8 — Proportion of the 200,000 run simulations in which each timber sample (columns) fell within the mean +/− one standard deviation of each source (rows). For all timber samples, the strontium shows uniform proportions across all possible sources. This indicates a high degree of uncertainty when attempting to use strontium to source timber samples. Only the San Mateo source emerges as a higher probability source for spruce (0.469) and fir (0.320). Original 87Sr/86Sr data were rounded to the 4th decimal place. (DOC) [file pone.0095580.s018.doc]

|  | Spruce | Fir | Ponderosa |
| --- | --- | --- | --- |
| Chuska Mountains | 0.150 | 0.122 | 0.144 |
| Chaco Watershed | 0.155 | 0.125 | 0.146 |
| Aztec Soil | 0.130 | 0.100 | 0.143 |
| San Mateo Mountains | 0.320 | 0.469 | 0.170 |
| San Pedro Mountains | 0.029 | 0.022 | 0.065 |
| La Plata Mountains | 0.110 | 0.080 | 0.135 |
| Hosta Butte | 0.074 | 0.055 | 0.122 |
| Cuba Mesa | 0.032 | 0.026 | 0.075 |
