# Supplementary material for: Strontium Isotopes and the Reconstruction of the Chaco Regional System: Evaluating Uncertainty with Bayesian Mixing Models
Source: PLoS One. 2014 May 22;9(5):e95580. doi: 10.1371/journal.pone.0095580 (PMC4031078; doi:10.1371/journal.pone.0095580)
Supplement: Table S9 — Maize Source Strontium Isotope Data. (DOC) [file pone.0095580.s019.doc]

| Sample site/no. | Site | Provenience | Depth | UTM Zone | UTM E | UTM N | 87Sr/86Sr | Error (2 SD) | Citation |
| --- | --- | --- | --- | --- | --- | --- | --- | --- | --- |
| AZR#1 | Aztec Ruin Soil | Animas River Bank | 22 to 27 | 12N | 768141 | 4080608 | 0.709899 | 0.00002 | Benson et al. 2009 |
| AZR#2 | Aztec Ruin Soil | Animas River Bank | 52 to 57 | 12N | 768141 | 4080608 | 0.709557 | 0.000017 | Benson et al. 2009 |
| AZR#3 | Aztec Ruin Soil | Aztec Ruin alluvial fan | 5 to 10 | 12N | 767969 | 4080757 | 0.709581 | 0.000013 | Benson et al. 2009 |
| AZR#4 | Aztec Ruin Soil | Aztec Ruin alluvial fan | 20 to 25 | 12N | 767969 | 4080757 | 0.70956 | 0.000021 | Benson et al. 2009 |
| AZR#5 | Aztec Ruin Soil | Aztec Ruin alluvial fan | 45 to 50 | 12N | 767969 | 4080757 | 0.709577 | 0.000019 | Benson et al. 2009 |
| AZR#6 | Aztec Ruin Soil | Aztec Ruin alluvial fan | 5 to 10 | 12N | 767483 | 4080660 | 0.70965 | 0.000011 | Benson et al. 2009 |
| AZR#7 | Aztec Ruin Soil | Aztec Ruin alluvial fan | 30 to 35 | 12N | 767483 | 4080660 | 0.7096 | 0.000017 | Benson et al. 2009 |
| AZR#8 | Aztec Ruin Soil | Aztec Ruin alluvial fan | 75 to 80 | 12N | 767483 | 4080660 | 0.709558 | 0.00002 | Benson et al. 2009 |
| AZR04-1 | Aztec Ruin Soil | Aztec Ruin floodplain | 45 to 50 | 12N | 768134 | 4080775 | 0.709573 | 0.000012 | Benson et al. 2009 |
| AZR04-2 | Aztec Ruin Soil | Aztec Ruin floodplain | 45 to 50 | 12N | 768122 | 4080682 | 0.709525 | 0.000031 | Benson et al. 2009 |
| AZR04-3 | Aztec Ruin Soil | Aztec Ruin floodplain | 45 to 50 | 12N | 768089 | 4080599 | 0.709587 | 0.000022 | Benson et al. 2009 |
| SAL#1 | Salmon Ruin Soil | Salmon Ruin Floodplain | 60 to 65 | 12N | 765623 | 4065336 | 0.71006 | 0.00002 | Benson et al. 2009 |
| SAL#2 | Salmon Ruin Soil | Salmon Ruin Floodplain | 55 to 60 | 12N | 765627 | 4065331 | 0.71001 | 0.00002 | Benson et al. 2009 |
| SJS#1 | Salmon Ruin Soil | San Juan River Bank @ Bloomfield | 45 to 50 | 12N | 769233 | 4065610 | 0.710237 | 0.000019 | Benson et al. 2009 |
| SR#1 | Salmon Ruin Soil | Salmon Ruin Orchard | 5 to 10 | 12N | 765635 | 4065725 | 0.710148 | 0.000013 | Benson et al. 2009 |
| SR#2 | Salmon Ruin Soil | Salmon Ruin Orchard | 20 to 25 | 12N | 765635 | 4065725 | 0.710157 | 0.000007 | Benson et al. 2009 |
| SR#3 | Salmon Ruin Soil | Salmon Ruin Orchard | 80 to 85 | 12N | 765635 | 4065725 | 0.710095 | 0.000023 | Benson et al. 2009 |
| SR#4 | Salmon Ruin Soil | Salmon Ruin Floodplain | 10 to 15 | 12N | 765658 | 4065573 | 0.710089 | 0.000019 | Benson et al. 2009 |
| SR#5 | Salmon Ruin Soil | Salmon Ruin Floodplain | 50 to 55 | 12N | 765658 | 4065573 | 0.710106 | 0.000022 | Benson et al. 2009 |
| SR#6 | Salmon Ruin Soil | Salmon Ruin Floodplain | 85 to 90 | 12N | 765658 | 4065573 | 0.710043 | 0.00002 | Benson et al. 2009 |
| HG06-1 | La Plata Soil | Holmes Grp. flood plain | 30 to 35 | 12N | 750842 | 4090876 | 0.709132 | 0.000014 | Benson et al. 2009 |
| HG06-2 | La Plata Soil | Holmes Grp. flood plain | 30 to 35 | 12N | 750836 | 4090858 | 0.70891 | 0.00001 | Benson et al. 2009 |
| HG06-3 | La Plata Soil | Holmes Grp. old flood plain | 30 to 35 | 12N | 750864 | 4090840 | 0.708829 | 0.00001 | Benson et al. 2009 |
| LP06-1 | La Plata Soil | La Plata R. flood plain | 30 to 35 | 12N | 750050 | 4097310 | 0.708767 | 0.000011 | Benson et al. 2009 |
| LP06-2 | La Plata Soil | La Plata R. flood plain | 30 to 35 | 12N | 750026 | 4097288 | 0.708837 | 0.00001 | Benson et al. 2009 |
| LP06-3 | La Plata Soil | La Plata R. old flood plain | 30 to 35 | 12N | 750080 | 4097259 | 0.70865 | 0.000013 | Benson et al. 2009 |
| CT#1 | Chuska Slope Soil | Captain Toms Wash | 95 to 100 | 12N | 702365 | 4016771 | 0.709289 | 0.000018 | Benson et al. 2009 |
| CT#2 | Chuska Slope Soil | Captain Toms Wash | 0 to 10 | 12N | 702574 | 4017016 | 0.709062 | 0.000023 | Benson et al. 2009 |
| CT#3 | Chuska Slope Soil | Captain Toms Wash | 0 to 10 | 12N | 706010 | 4020290 | 0.709028 | 0.000017 | Benson et al. 2009 |
| CTW#1A | Chuska Slope Soil | Captain Toms Wash | 25 to 35 | 12N | 702364 | 4016768 | 0.709018 | 0.000009 | Benson et al. 2009 |
| CTW#1B | Chuska Slope Soil | Captain Toms Wash | 55 to 65 | 12N | 702364 | 4016768 | 0.70924 | 0.000012 | Benson et al. 2009 |
| CTW#1C | Chuska Slope Soil | Captain Toms Wash | 85 to 95 | 12N | 702364 | 4016768 | 0.70929 | 0.000017 | Benson et al. 2009 |
| CTW#2A | Chuska Slope Soil | Captain Toms Wash | 25 to 35 | 12N | 702569 | 4017023 | 0.709046 | 0.000009 | Benson et al. 2009 |
| CTW#2B | Chuska Slope Soil | Captain Toms Wash | 55 to 65 | 12N | 702569 | 4017023 | 0.709215 | 0.000015 | Benson et al. 2009 |
| CTW#2C | Chuska Slope Soil | Captain Toms Wash | 85 to 95 | 12N | 702569 | 4017023 | 0.709083 | 0.000019 | Benson et al. 2009 |
| CTW#3A | Chuska Slope Soil | Captain Toms Wash | 25 to 35 | 12N | 705994 | 4020307 | 0.70883 | 0.000021 | Benson et al. 2009 |
| CTW#3B | Chuska Slope Soil | Captain Toms Wash | 55 to 65 | 12N | 705994 | 4020307 | 0.70876 | 0.000008 | Benson et al. 2009 |
| CTW#3C | Chuska Slope Soil | Captain Toms Wash | 85 to 95 | 12N | 705994 | 4020307 | 0.708709 | 0.000016 | Benson et al. 2009 |
| SA06-1 | Chuska Slope Soil | Sanostee | 30 to 35 | 12N | 682870 | 4033219 | 0.708504 | 0.00001 | Benson et al. 2009 |
| SA06-2 | Chuska Slope Soil | Sanostee | 30 to 35 | 12N | 682785 | 4033136 | 0.708743 | 0.000007 | Benson et al. 2009 |
| SA06-3 | Chuska Slope Soil | Sanostee | 30 to 35 | 12N | 689406 | 4033361 | 0.708971 | 0.000009 | Benson et al. 2009 |
| TO06-1 | Chuska Slope Soil | Tocito seep | 30 to 35 | 12N | 698913 | 4030608 | 0.708765 | 0.000013 | Benson et al. 2009 |
| TO06-2 | Chuska Slope Soil | Tocito Wash | 30 to 35 | 12N | 698096 | 4025821 | 0.708895 | 0.000012 | Benson et al. 2009 |
| SS#1 | Chuska Slope Soil | Skunk Springs Bank Deposit | 95 to 100 | 12N | 701539 | 4011733 | 0.709331 | 0.000022 | Benson et al. 2009 |
| SS#2 | Chuska Slope Soil | Skunk Springs Modern Sediments | 0 to 5 | 12N | 701539 | 4011733 | 0.709119 | 0.00002 | Benson et al. 2009 |
| SS#3 | Chuska Slope Soil | Skunk Springs Wash Edge | 105 to 110 | 12N | 701539 | 4011733 | 0.709349 | 0.000022 | Benson et al. 2009 |
| TGHBM#1 | Chuska Slope Soil | Two Grey Hills Basketmaker | 70 to 75 | 12N | 695786 | 4011891 | 0.70904 | 0.00002 | Benson et al. 2009 |
| TGHBM#2 | Chuska Slope Soil | Two Grey Hills Basketmaker | 80 to 85 | 12N | 695816 | 4011920 | 0.70917 | 0.00002 | Benson et al. 2009 |
| TGHBM#3 | Chuska Slope Soil | Two Grey Hills Basketmaker | 65 to 70 | 12N | 695619 | 4012007 | 0.70912 | 0.00002 | Benson et al. 2009 |
| TGHBM#4 | Chuska Slope Soil | Two Grey Hills Basketmaker | 70 to 75 | 12N | 695965 | 4011503 | 0.70923 | 0.00002 | Benson et al. 2009 |
| TGHBM#5 | Chuska Slope Soil | Two Grey Hills Basketmaker | 55 to 60 | 12N | 695631 | 4011075 | 0.7092 | 0.00002 | Benson et al. 2009 |
| TGHBM#6 | Chuska Slope Soil | Two Grey Hills Basketmaker | 65 to 70 | 12N | 695131 | 4010586 | 0.7093 | 0.00002 | Benson et al. 2009 |
| TGHBM#7 | Chuska Slope Soil | Two Grey Hills Basketmaker | 65 to 70 | 12N | 693258 | 4010745 | 0.70906 | 0.00002 | Benson et al. 2009 |
| BTW07-1 | Chaco Watershed | Betonnie Tsasie Wash | 30 to 35 | 12N | 797613 | 4011438 | 0.709462 | 0.000014 | Benson et al. 2009 |
| BI06-1 | Chaco Watershed | Bis sa ani | 30 to 35 | 12N | 786575 | 3996889 | 0.708713 | 0.000013 | Benson et al. 2009 |
| BI06-2 | Chaco Watershed | Bis sa ani | 30 to 35 | 12N | 787530 | 3997645 | 0.70898 | 0.000009 | Benson et al. 2009 |
| CDR04-1 | Chaco Watershed | Casa del Rio | 50 to 55 | 12N | 762573 | 3998249 | 0.709285 | 0.000015 | Benson et al. 2009 |
| CDR04-2 | Chaco Watershed | Casa del Rio | 50 to 55 | 12N | 762832 | 3997950 | 0.70905 | 0.000017 | Benson et al. 2009 |
| CDR04-3 | Chaco Watershed | Casa del Rio | 50 to 55 | 12N | 762772 | 3997745 | 0.709354 | 0.00002 | Benson et al. 2009 |
| CDR04-4 | Chaco Watershed | Casa del Rio | 50 to 55 | 12N | 764131 | 3995670 | 0.709104 | 0.000021 | Benson et al. 2009 |
| CW371-04-1 | Chaco Watershed | Chaco Wash @ HWY 371 | 45 to 50 | 12N | 752926 | 3999894 | 0.708888 | 0.000028 | Benson et al. 2009 |
| EC06-1 | Chaco Watershed | East Community | 30 to 35 | 12N | 789899 | 3987337 | 0.709089 | 0.000011 | Benson et al. 2009 |
| EC06-2 | Chaco Watershed | East Community | 30 to 35 | 12N | 787820 | 3987924 | 0.70906 | 0.000013 | Benson et al. 2009 |
| EC06-3 | Chaco Watershed | East Community | 30 to 35 | 12N | 786309 | 3989300 | 0.709049 | 0.000012 | Benson et al. 2009 |
| EC06-4 | Chaco Watershed | East Community | 30 to 35 | 12N | 792914 | 3986504 | 0.709499 | 0.00001 | Benson et al. 2009 |
| EC06-5 | Chaco Watershed | East Community | 30 to 35 | 12N | 792950 | 3986951 | 0.709593 | 0.000013 | Benson et al. 2009 |
| EC06-6 | Chaco Watershed | East Community, flood plain | 30 to 35 | 12N | 792398 | 3987501 | 0.709126 | 0.000009 | Benson et al. 2009 |
| ES04-1 | Chaco Watershed | Escalon | 45 to 50 | 12N | 736928 | 4000890 | 0.709637 | 0.000017 | Benson et al. 2009 |
| ES04-2 | Chaco Watershed | Escalon | 45 to 50 | 12N | 737206 | 4002189 | 0.709489 | 0.000018 | Benson et al. 2009 |
| EW04-1 | Chaco Watershed | Escavada Wash | 45 to 50 | 12N | 774001 | 3999476 | 0.709073 | 0.000028 | Benson et al. 2009 |
| GB04-1 | Chaco Watershed | Great Bend | 45 to 50 | 12N | 727046 | 4004855 | 0.709557 | 0.000012 | Benson et al. 2009 |
| GB04-2 | Chaco Watershed | Great Bend | 45 to 50 | 12N | 725178 | 4005576 | 0.709408 | 0.000019 | Benson et al. 2009 |
| KW07-1 | Chaco Watershed | Kimbeto Wash | 30 to 35 | 12N | 794485 | 4014947 | 0.709243 | 0.000011 | Benson et al. 2009 |
| KB04-1 | Chaco Watershed | Kin Bineola | 45 to 50 | 12N | 757669 | 3987748 | 0.709438 | 0.000013 | Benson et al. 2009 |
| KB04-2 | Chaco Watershed | Kin Bineola | 45 to 50 | 12N | 757663 | 3987602 | 0.70931 | 0.000012 | Benson et al. 2009 |
| KB04-3 | Chaco Watershed | Kin Bineola | 45 to 50 | 12N | 757633 | 3987480 | 0.709324 | 0.000012 | Benson et al. 2009 |
| KBV04-1 | Chaco Watershed | Kin Bineola Valley | 50 to 55 | 12N | 757222 | 3990342 | 0.709627 | 0.00002 | Benson et al. 2009 |
| KK04-1 | Chaco Watershed | Kin Klizhin | 45 to 50 | 12N | 763797 | 3991159 | 0.709527 | 0.000011 | Benson et al. 2009 |
| KK04-2 | Chaco Watershed | Kin Klizhin | 45 to 50 | 12N | 763995 | 3991388 | 0.709474 | 0.000016 | Benson et al. 2009 |
| PP04-1 | Chaco Watershed | Pueblo Pintado | 45 to 50 | 12N | 798823 | 3987422 | 0.709199 | 0.000018 | Benson et al. 2009 |
| PP04-2 | Chaco Watershed | Pueblo Pintado | 45 to 50 | 12N | 798565 | 3987448 | 0.709318 | 0.000025 | Benson et al. 2009 |
| PP04-3 | Chaco Watershed | Pueblo Pintado | 50 to 55 | 12N | 798715 | 3987071 | 0.709615 | 0.000018 | Benson et al. 2009 |
| PP04-4 | Chaco Watershed | Pueblo Pintado | 50 to 55 | 12N | 799009 | 3986866 | 0.708868 | 0.000015 | Benson et al. 2009 |
| PP04-5 | Chaco Watershed | Pueblo Pintado | 50 to 55 | 12N | 800595 | 3985762 | 0.70941 | 0.000017 | Benson et al. 2009 |
| RR06-1 | Chaco Watershed | Reservoir Ruin | 30 to 35 | 12N | 809967 | 3972878 | 0.709554 | 0.000008 | Benson et al. 2009 |
| RR06-2 | Chaco Watershed | Reservoir Ruin | 30 to 35 | 12N | 809961 | 3972708 | 0.709673 | 0.000013 | Benson et al. 2009 |
| RW06-1 | Chaco Watershed | Raton Well | 30 to 35 | 12N | 813467 | 3979455 | 0.708769 | 0.000014 | Benson et al. 2009 |
| RW06-2 | Chaco Watershed | Raton Well | 30 to 35 | 12N | 813543 | 3979514 | 0.708905 | 0.000011 | Benson et al. 2009 |
| SR06-1 | Chaco Watershed | Shepard Ruin | 30 to 35 | 12N | 806649 | 3978080 | 0.709271 | 0.000009 | Benson et al. 2009 |
| WC04-1 | Chaco Watershed | Willow Canyon | 45 to 50 | 12N | 732062 | 4002278 | 0.709547 | 0.000013 | Benson et al. 2009 |
| WC04-2 | Chaco Watershed | Willow Canyon | 45 to 50 | 12N | 731985 | 4002361 | 0.709503 | 0.000015 | Benson et al. 2009 |
| WF06-1 | Chaco Watershed | Windmill Facility | 30 to 35 | 12N | 804055 | 3985268 | 0.709237 | 0.000012 | Benson et al. 2009 |
| CC#1 | Chaco Watershed | Casa Chiquita | 67 to 72 | 12N | 772601 | 3995237 | 0.709036 | 0.000013 | Benson et al. 2009 |
| CC#2 | Chaco Watershed | Casa Chiquita | 97 to 102 | 12N | 772601 | 3995237 | 0.709083 | 0.000015 | Benson et al. 2009 |
| CC#3 | Chaco Watershed | Casa Chiquita | 127 to 132 | 12N | 772601 | 3995237 | 0.709066 | 0.000011 | Benson et al. 2009 |
| CK#1 | Chaco Watershed | Chetro Ketl Field | 5 to 10 | 12N | 774673 | 3994588 | 0.70919 | 0.000017 | Benson et al. 2009 |
| CKF#1 | Chaco Watershed | Chetro Ketl Field | 25 to 35 | 12N | 774683 | 3994453 | 0.70917 | 0.000012 | Benson et al. 2009 |
| CKF#2 | Chaco Watershed | Chetro Ketl Field | 55 to 65 | 12N | 774683 | 3994453 | 0.709065 | 0.000015 | Benson et al. 2009 |
| CKF#3 | Chaco Watershed | Chetro Ketl Field | 85 to 95 | 12N | 774683 | 3994453 | 0.709053 | 0.000009 | Benson et al. 2009 |
| CR#1 | Chaco Watershed | Casa Rinconada | 25 to 35 | 12N | 773816 | 3994289 | 0.709159 | 0.000009 | Benson et al. 2009 |
| CR#2 | Chaco Watershed | Casa Rinconada | 55 to 65 | 12N | 773816 | 3994289 | 0.709088 | 0.000017 | Benson et al. 2009 |
| CR#3 | Chaco Watershed | Casa Rinconada | 85 to 95 | 12N | 773816 | 3994289 | 0.709108 | 0.000017 | Benson et al. 2009 |
| FB04-1 | Chaco Watershed | Fajada Butte | 45 to 50 | 12N | 778168 | 3990671 | 0.708973 | 0.000014 | Benson et al. 2009 |
| FB04-2 | Chaco Watershed | Fajada Butte | 45 to 50 | 12N | 778101 | 3990753 | 0.709005 | 0.000018 | Benson et al. 2009 |
| LH#1 | Chaco Watershed | Lizard House Arroyo | 25 to 30 | 12N | 774970 | 3994402 | 0.708965 | 0.000015 | Benson et al. 2009 |
| LH#2 | Chaco Watershed | Lizard House Arroyo | 62 to 67 | 12N | 774970 | 3994402 | 0.709347 | 0.00002 | Benson et al. 2009 |
| LH#3 | Chaco Watershed | Lizard House Arroyo | 145 to 150 | 12N | 774970 | 3994402 | 0.709165 | 0.000013 | Benson et al. 2009 |
| PDA#1 | Chaco Watershed | Pueblo del Arroyo | 435 to 445 | 12N | 773188 | 3994969 | 0.709077 | 0.000023 | Benson et al. 2009 |
| PDA#3 | Chaco Watershed | Pueblo del Arroyo | 320 to 330 | 12N | 773188 | 3994969 | 0.709155 | 0.000017 | Benson et al. 2009 |
| PDA#4 | Chaco Watershed | Pueblo del Arroyo | 15 to 25 | 12N | 773188 | 3994969 | 0.709093 | 0.000014 | Benson et al. 2009 |
| PDA#5 | Chaco Watershed | Pueblo del Arroyo | 0 to 3 | 12N | 773158 | 3994925 | 0.709044 | 0.000017 | Benson et al. 2009 |
| S10#1 | Chaco Watershed | Penasco Blanco Field | 25 to 35 | 12N | 771485 | 3996044 | 0.709204 | 0.000015 | Benson et al. 2009 |
| S10#2 | Chaco Watershed | Penasco Blanco Field | 55 to 65 | 12N | 771485 | 3996044 | 0.709121 | 0.000017 | Benson et al. 2009 |
| S10#3 | Chaco Watershed | Penasco Blanco Field | 85 to 95 | 12N | 771485 | 3996044 | 0.709078 | 0.000012 | Benson et al. 2009 |
| WER#1 | Chaco Watershed | Weritos Rincon | 25 to 35 | 12N | 775694 | 3993419 | 0.70959 | 0.00001 | Benson et al. 2009 |
| WER#2 | Chaco Watershed | Weritos Rincon | 55 to 65 | 12N | 775694 | 3993419 | 0.709606 | 0.000018 | Benson et al. 2009 |
| WER#3 | Chaco Watershed | Weritos Rincon | 85 to 95 | 12N | 775694 | 3993419 | 0.709549 | 0.000001 | Benson et al. 2009 |
| WR#1 | Chaco Watershed | Weritos Rincon | 0 to 10 | 12N | 775710 | 3993536 | 0.70957 | 0.000021 | Benson et al. 2009 |
| WR#2 | Chaco Watershed | Weritos Rincon | 0 to 10 | 12N | 775651 | 3993412 | 0.709465 | 0.000008 | Benson et al. 2009 |
| CC04-1 | Chaco Watershed | Clys Canyon | 45 to 50 | 12N | 772738 | 3995822 | 0.708792 | 0.000022 | Benson et al. 2009 |
| GW04-1 | Chaco Watershed | Gallo Wash | 45 to 50 | 12N | 782121 | 3993794 | 0.709311 | 0.000014 | Benson et al. 2009 |
| GW04-2 | Chaco Watershed | Gallo Wash | 45 to 50 | 12N | 781016 | 3992867 | 0.708963 | 0.000018 | Benson et al. 2009 |
| GW04-3 | Chaco Watershed | Gallo Wash | 45 to 50 | 12N | 781056 | 3992821 | 0.708996 | 0.000012 | Benson et al. 2009 |
| MC04-1 | Chaco Watershed | Mockingbird Canyon | 45 to 50 | 12N | 777227 | 3993962 | 0.709144 | 0.000013 | Benson et al. 2009 |
| MC04-2 | Chaco Watershed | Mockingbird Canyon | 45 to 50 | 12N | 777075 | 3993897 | 0.709208 | 0.000019 | Benson et al. 2009 |
| SG04-1 | Chaco Watershed | South Gap | 45 to 50 | 12N | 772784 | 3993144 | 0.709421 | 0.00002 | Benson et al. 2009 |
| SG04-2 | Chaco Watershed | South Gap | 45 to 50 | 12N | 772898 | 3993453 | 0.709617 | 0.000029 | Benson et al. 2009 |
| SG04-3 | Chaco Watershed | South Gap | 45 to 50 | 12N | 773006 | 3993777 | 0.709709 | 0.00002 | Benson et al. 2009 |
| KY04-1 | Lobo Mesa Soil | Kin Ya-a | 45 to 50 | 12N | 761678 | 3951444 | 0.709462 | 0.000027 | Benson et al. 2009 |
| KY04-2 | Lobo Mesa Soil | Kin Ya-a | 45 to 50 | 12N | 761533 | 3950674 | 0.709737 | 0.000022 | Benson et al. 2009 |
| PS04-1 | Lobo Mesa Soil | Peach Spring | 50 to 55 | 12N | 723388 | 3963613 | 0.709863 | 0.000011 | Benson et al. 2009 |
| RW04-1 | Lobo Mesa Soil | Red Willow | 50 to 55 | 12N | 708197 | 3967829 | 0.709871 | 0.000025 | Benson et al. 2009 |
| RW04-2 | Lobo Mesa Soil | Red Willow | 50 to 55 | 12N | 709759 | 3967743 | 0.710125 | 0.000026 | Benson et al. 2009 |
| SR04-1 | Lobo Mesa Soil | Standing Rock | 50 to 55 | 12N | 735795 | 3967510 | 0.709853 | 0.000019 | Benson et al. 2009 |
| AC04-1 | Red Mesa Soil | Andrews Community | 50 to 55 | 12N | 770021 | 3917460 | 0.709306 | 0.000018 | Benson et al. 2009 |
| HS04-1 | Red Mesa Soil | Haystack | 50 to 55 | 12N | 782836 | 3915027 | 0.708577 | 0.000019 | Benson et al. 2009 |
| SM04-1 | Red Mesa Soil | San Mateo | 50 to 55 | 12N | 804631 | 3915902 | 0.709188 | 0.000018 | Benson et al. 2009 |
| SM04-2 | Red Mesa Soil | San Mateo | 50 to 55 | 12N | 804152 | 3915295 | 0.708909 | 0.000017 | Benson et al. 2009 |
| SM04-3 | Red Mesa Soil | San Mateo | 50 to 55 | 12N | 804318 | 3915929 | 0.709042 | 0.000014 | Benson et al. 2009 |
| MR07-1 | N San Juan River Trib | Mancos River floodplain | 30 to 35 | 12N | 700889 | 4100093 | 0.708697 | 0.000013 | Benson et al. 2009 |
| NW07-1 | N San Juan River Trib | Navajo Wash incised floodplain | 30 to 35 | 12N | 700014 | 4106099 | 0.708226 | 0.000011 | Benson et al. 2009 |
| MAW07-1 | N San Juan River Trib | Marble Wash floodplain | 30 to 35 | 12N | 673356 | 4113451 | 0.708952 | 0.00001 | Benson et al. 2009 |
| McC07-1 | N San Juan River Trib | McElmo Creek floodplain | 30 to 35 | 12N | 660602 | 4120530 | 0.708785 | 0.000023 | Benson et al. 2009 |
| MOC07-1 | N San Juan River Trib | Montezuma Creek floodplain | 30 to 35 | 12N | 648181 | 4126168 | 0.709044 | 0.000014 | Benson et al. 2009 |
| REW07-1 | N San Juan River Trib | Recapture Wash floodplain | 30 to 35 | 12N | 636661 | 4129002 | 0.708702 | 0.000008 | Benson et al. 2009 |
| COW07-1 | N San Juan River Trib | Cottonwood Wash floodplain | 30 to 35 | 12N | 627492 | 4127038 | 0.708762 | 0.000013 | Benson et al. 2009 |
| CW07-1 | N San Juan River Trib | Combs Wash floodplain | 30 to 35 | 12N | 617537 | 4125049 | 0.708894 | 0.000014 | Benson et al. 2009 |
| BW07-1 | N San Juan River Trib | Butler Wash floodplain | 30 to 35 | 12N | 619520 | 4124715 | 0.708907 | 0.000014 | Benson et al. 2009 |
| SJR07-1 | N San Juan River Trib | San Juan River floodplain | 30 to 35 | 12N | 622367 | 4124066 | 0.708968 | 0.000014 | Benson et al. 2009 |
| CHW07-1 | N San Juan River Trib | Chinle Wash floodplain | 30 to 35 | 12N | 614932 | 4089320 | 0.708721 | 0.000013 | Benson et al. 2009 |
| WW07-1 | N San Juan River Trib | Walker Wash floodplain | 30 to 35 | 12N | 624256 | 4087136 | 0.708525 | 0.000007 | Benson et al. 2009 |
| TNP07-1 | N San Juan River Trib | Teec Nos Pos Wash floodplain | 30 to 35 | 12N | 668352 | 4088915 | 0.708329 | 0.000013 | Benson et al. 2009 |
| RW07-1 | N San Juan River Trib | Red Wash floodplain | 30 to 35 | 12N | 683824 | 4077492 | 0.708226 | 0.000012 | Benson et al. 2009 |
| SRW07-1 | N San Juan River Trib | Shiprock Wash floodplain | 30 to 35 | 12N | 693319 | 4073746 | 0.708243 | 0.000014 | Benson et al. 2009 |
