# Supplementary material for: Strontium Isotopes and the Reconstruction of the Chaco Regional System: Evaluating Uncertainty with Bayesian Mixing Models
Source: PLoS One. 2014 May 22;9(5):e95580. doi: 10.1371/journal.pone.0095580 (PMC4031078; doi:10.1371/journal.pone.0095580)
Supplement: Table S10 — Tree Source Strontium Isotope Data. (DOC) [file pone.0095580.s020.doc]

| Sample Name | Site | Site Name | Taxa | UTM Zone | UTM E | UTM N | 87 Sr/86 Sr corrected | 87 Sr/86 Sr uncorrected | Error (SD) | Citation |
| --- | --- | --- | --- | --- | --- | --- | --- | --- | --- | --- |
| BTW07-1 | Betonnie Tsasie Wash | Chaco Watershed |  | 12S | 797613 | 4011438 | 0.709462 | 0.709462 | 0.000014 | Benson et al 2009 |
| BI06-1 | Bis sa ani | Chaco Watershed |  | 12S | 786575 | 3996889 | 0.708713 | 0.708713 | 0.000013 | Benson et al 2009 |
| BI06-2 | Bis sa ani | Chaco Watershed |  | 12S | 787530 | 3997645 | 0.70898 | 0.70898 | 0.000009 | Benson et al 2009 |
| CDR04-1 | Casa del Rio | Chaco Watershed |  | 12S | 762573 | 3998249 | 0.709285 | 0.709285 | 0.000015 | Benson et al 2009 |
| CDR04-2 | Casa del Rio | Chaco Watershed |  | 12S | 762832 | 3997950 | 0.70905 | 0.70905 | 0.000017 | Benson et al 2009 |
| CDR04-3 | Casa del Rio | Chaco Watershed |  | 12S | 762772 | 3997745 | 0.709354 | 0.709354 | 0.00002 | Benson et al 2009 |
| CDR04-4 | Casa del Rio | Chaco Watershed |  | 12S | 764131 | 3995670 | 0.709104 | 0.709104 | 0.000021 | Benson et al 2009 |
| CW371-04-1 | Chaco Wash @ HWY 371 | Chaco Watershed |  | 12S | 752926 | 3999894 | 0.708888 | 0.708888 | 0.000028 | Benson et al 2009 |
| EC06-1 | East Community | Chaco Watershed |  | 12S | 789899 | 3987337 | 0.709089 | 0.709089 | 0.000011 | Benson et al 2009 |
| EC06-2 | East Community | Chaco Watershed |  | 12S | 787820 | 3987924 | 0.70906 | 0.70906 | 0.000013 | Benson et al 2009 |
| EC06-3 | East Community | Chaco Watershed |  | 12S | 786309 | 3989300 | 0.709049 | 0.709049 | 0.000012 | Benson et al 2009 |
| EC06-4 | East Community | Chaco Watershed |  | 12S | 792914 | 3986504 | 0.709499 | 0.709499 | 0.00001 | Benson et al 2009 |
| EC06-5 | East Community | Chaco Watershed |  | 12S | 792950 | 3986951 | 0.709593 | 0.709593 | 0.000013 | Benson et al 2009 |
| EC06-6 | East Community, flood plain | Chaco Watershed |  | 12S | 792398 | 3987501 | 0.709126 | 0.709126 | 0.000009 | Benson et al 2009 |
| ES04-1 | Escalon | Chaco Watershed |  | 12S | 736928 | 4000890 | 0.709637 | 0.709637 | 0.000017 | Benson et al 2009 |
| ES04-2 | Escalon | Chaco Watershed |  | 12S | 737206 | 4002189 | 0.709489 | 0.709489 | 0.000018 | Benson et al 2009 |
| EW04-1 | Escavada Wash | Chaco Watershed |  | 12S | 774001 | 3999476 | 0.709073 | 0.709073 | 0.000028 | Benson et al 2009 |
| GB04-1 | Great Bend | Chaco Watershed |  | 12S | 727046 | 4004855 | 0.709557 | 0.709557 | 0.000012 | Benson et al 2009 |
| GB04-2 | Great Bend | Chaco Watershed |  | 12S | 725178 | 4005576 | 0.709408 | 0.709408 | 0.000019 | Benson et al 2009 |
| KW07-1 | Kimbeto Wash | Chaco Watershed |  | 12S | 794485 | 4014947 | 0.709243 | 0.709243 | 0.000011 | Benson et al 2009 |
| KB04-1 | Kin Bineola | Chaco Watershed |  | 12S | 757669 | 3987748 | 0.709438 | 0.709438 | 0.000013 | Benson et al 2009 |
| KB04-2 | Kin Bineola | Chaco Watershed |  | 12S | 757663 | 3987602 | 0.70931 | 0.70931 | 0.000012 | Benson et al 2009 |
| KB04-3 | Kin Bineola | Chaco Watershed |  | 12S | 757633 | 3987480 | 0.709324 | 0.709324 | 0.000012 | Benson et al 2009 |
| KBV04-1 | Kin Bineola Valley | Chaco Watershed |  | 12S | 757222 | 3990342 | 0.709627 | 0.709627 | 0.00002 | Benson et al 2009 |
| KK04-1 | Kin Klizhin | Chaco Watershed |  | 12S | 763797 | 3991159 | 0.709527 | 0.709527 | 0.000011 | Benson et al 2009 |
| KK04-2 | Kin Klizhin | Chaco Watershed |  | 12S | 763995 | 3991388 | 0.709474 | 0.709474 | 0.000016 | Benson et al 2009 |
| PP04-1 | Pueblo Pintado | Chaco Watershed |  | 12S | 798823 | 3987422 | 0.709199 | 0.709199 | 0.000018 | Benson et al 2009 |
| PP04-2 | Pueblo Pintado | Chaco Watershed |  | 12S | 798565 | 3987448 | 0.709318 | 0.709318 | 0.000025 | Benson et al 2009 |
| PP04-3 | Pueblo Pintado | Chaco Watershed |  | 12S | 798715 | 3987071 | 0.709615 | 0.709615 | 0.000018 | Benson et al 2009 |
| PP04-4 | Pueblo Pintado | Chaco Watershed |  | 12S | 799009 | 3986866 | 0.708868 | 0.708868 | 0.000015 | Benson et al 2009 |
| PP04-5 | Pueblo Pintado | Chaco Watershed |  | 12S | 800595 | 3985762 | 0.70941 | 0.70941 | 0.000017 | Benson et al 2009 |
| RR06-1 | Reservoir Ruin | Chaco Watershed |  | 12S | 809967 | 3972878 | 0.709554 | 0.709554 | 0.000008 | Benson et al 2009 |
| RR06-2 | Reservoir Ruin | Chaco Watershed |  | 12S | 809961 | 3972708 | 0.709673 | 0.709673 | 0.000013 | Benson et al 2009 |
| RW06-1 | Raton Well | Chaco Watershed |  | 12S | 813467 | 3979455 | 0.708769 | 0.708769 | 0.000014 | Benson et al 2009 |
| RW06-2 | Raton Well | Chaco Watershed |  | 12S | 813543 | 3979514 | 0.708905 | 0.708905 | 0.000011 | Benson et al 2009 |
| SR06-1 | Shepard Ruin | Chaco Watershed |  | 12S | 806649 | 3978080 | 0.709271 | 0.709271 | 0.000009 | Benson et al 2009 |
| WC04-1 | Willow Canyon | Chaco Watershed |  | 12S | 732062 | 4002278 | 0.709547 | 0.709547 | 0.000013 | Benson et al 2009 |
| WC04-2 | Willow Canyon | Chaco Watershed |  | 12S | 731985 | 4002361 | 0.709503 | 0.709503 | 0.000015 | Benson et al 2009 |
| WF06-1 | Windmill Facility | Chaco Watershed |  | 12S | 804055 | 3985268 | 0.709237 | 0.709237 | 0.000012 | Benson et al 2009 |
| CC#1 | Casa Chiquita | Chaco Watershed |  | 12S | 772601 | 3995237 | 0.709036 | 0.709036 | 0.000013 | Benson et al 2009 |
| CC#2 | Casa Chiquita | Chaco Watershed |  | 12S | 772601 | 3995237 | 0.709083 | 0.709083 | 0.000015 | Benson et al 2009 |
| CC#3 | Casa Chiquita | Chaco Watershed |  | 12S | 772601 | 3995237 | 0.709066 | 0.709066 | 0.000011 | Benson et al 2009 |
| CK#1 | Chetro Ketl Field | Chaco Watershed |  | 12S | 774673 | 3994588 | 0.70919 | 0.70919 | 0.000017 | Benson et al 2009 |
| CKF#1 | Chetro Ketl Field | Chaco Watershed |  | 12S | 774683 | 3994453 | 0.70917 | 0.70917 | 0.000012 | Benson et al 2009 |
| CKF#2 | Chetro Ketl Field | Chaco Watershed |  | 12S | 774683 | 3994453 | 0.709065 | 0.709065 | 0.000015 | Benson et al 2009 |
| CKF#3 | Chetro Ketl Field | Chaco Watershed |  | 12S | 774683 | 3994453 | 0.709053 | 0.709053 | 0.000009 | Benson et al 2009 |
| CR#1 | Casa Rinconada | Chaco Watershed |  | 12S | 773816 | 3994289 | 0.709159 | 0.709159 | 0.000009 | Benson et al 2009 |
| CR#2 | Casa Rinconada | Chaco Watershed |  | 12S | 773816 | 3994289 | 0.709088 | 0.709088 | 0.000017 | Benson et al 2009 |
| CR#3 | Casa Rinconada | Chaco Watershed |  | 12S | 773816 | 3994289 | 0.709108 | 0.709108 | 0.000017 | Benson et al 2009 |
| FB04-1 | Fajada Butte | Chaco Watershed |  | 12S | 778168 | 3990671 | 0.708973 | 0.708973 | 0.000014 | Benson et al 2009 |
| FB04-2 | Fajada Butte | Chaco Watershed |  | 12S | 778101 | 3990753 | 0.709005 | 0.709005 | 0.000018 | Benson et al 2009 |
| LH#1 | Lizard House Arroyo | Chaco Watershed |  | 12S | 774970 | 3994402 | 0.708965 | 0.708965 | 0.000015 | Benson et al 2009 |
| LH#2 | Lizard House Arroyo | Chaco Watershed |  | 12S | 774970 | 3994402 | 0.709347 | 0.709347 | 0.00002 | Benson et al 2009 |
| LH#3 | Lizard House Arroyo | Chaco Watershed |  | 12S | 774970 | 3994402 | 0.709165 | 0.709165 | 0.000013 | Benson et al 2009 |
| PDA#1 | Pueblo del Arroyo | Chaco Watershed |  | 12S | 773188 | 3994969 | 0.709077 | 0.709077 | 0.000023 | Benson et al 2009 |
| PDA#3 | Pueblo del Arroyo | Chaco Watershed |  | 12S | 773188 | 3994969 | 0.709155 | 0.709155 | 0.000017 | Benson et al 2009 |
| PDA#4 | Pueblo del Arroyo | Chaco Watershed |  | 12S | 773188 | 3994969 | 0.709093 | 0.709093 | 0.000014 | Benson et al 2009 |
| PDA#5 | Pueblo del Arroyo | Chaco Watershed |  | 12S | 773158 | 3994925 | 0.709044 | 0.709044 | 0.000017 | Benson et al 2009 |
| S10#1 | Penasco Blanco Field | Chaco Watershed |  | 12S | 771485 | 3996044 | 0.709204 | 0.709204 | 0.000015 | Benson et al 2009 |
| S10#2 | Penasco Blanco Field | Chaco Watershed |  | 12S | 771485 | 3996044 | 0.709121 | 0.709121 | 0.000017 | Benson et al 2009 |
| S10#3 | Penasco Blanco Field | Chaco Watershed |  | 12S | 771485 | 3996044 | 0.709078 | 0.709078 | 0.000012 | Benson et al 2009 |
| WER#1 | Weritos Rincon | Chaco Watershed |  | 12S | 775694 | 3993419 | 0.70959 | 0.70959 | 0.00001 | Benson et al 2009 |
| WER#2 | Weritos Rincon | Chaco Watershed |  | 12S | 775694 | 3993419 | 0.709606 | 0.709606 | 0.000018 | Benson et al 2009 |
| WER#3 | Weritos Rincon | Chaco Watershed |  | 12S | 775694 | 3993419 | 0.709549 | 0.709549 | 0.000001 | Benson et al 2009 |
| WR#1 | Weritos Rincon | Chaco Watershed |  | 12S | 775710 | 3993536 | 0.70957 | 0.70957 | 0.000021 | Benson et al 2009 |
| WR#2 | Weritos Rincon | Chaco Watershed |  | 12S | 775651 | 3993412 | 0.709465 | 0.709465 | 0.000008 | Benson et al 2009 |
| CC04-1 | Clys Canyon | Chaco Watershed |  | 12S | 772738 | 3995822 | 0.708792 | 0.708792 | 0.000022 | Benson et al 2009 |
| GW04-1 | Gallo Wash | Chaco Watershed |  | 12S | 782121 | 3993794 | 0.709311 | 0.709311 | 0.000014 | Benson et al 2009 |
| GW04-2 | Gallo Wash | Chaco Watershed |  | 12S | 781016 | 3992867 | 0.708963 | 0.708963 | 0.000018 | Benson et al 2009 |
| GW04-3 | Gallo Wash | Chaco Watershed |  | 12S | 781056 | 3992821 | 0.708996 | 0.708996 | 0.000012 | Benson et al 2009 |
| MC04-1 | Mockingbird Canyon | Chaco Watershed |  | 12S | 777227 | 3993962 | 0.709144 | 0.709144 | 0.000013 | Benson et al 2009 |
| MC04-2 | Mockingbird Canyon | Chaco Watershed |  | 12S | 777075 | 3993897 | 0.709208 | 0.709208 | 0.000019 | Benson et al 2009 |
| SG04-1 | South Gap | Chaco Watershed |  | 12S | 772784 | 3993144 | 0.709421 | 0.709421 | 0.00002 | Benson et al 2009 |
| SG04-2 | South Gap | Chaco Watershed |  | 12S | 772898 | 3993453 | 0.709617 | 0.709617 | 0.000029 | Benson et al 2009 |
| SG04-3 | South Gap | Chaco Watershed |  | 12S | 773006 | 3993777 | 0.709709 | 0.709709 | 0.00002 | Benson et al 2009 |
| CKAMT-66 |  | Chuska Mountains | Abieslasiocarpa | 12N | 689466 | 4017841 | 0.7097974 | 0.7097627 | 0.000015 | English et al. 2001 |
| CKAMT-67 |  | Chuska Mountains | A. lasiocarpa | 12N | 689466 | 4017841 | 0.7097881 | 0.7097534 | 0.000011 | English et al. 2001 |
| CKAMT-69 |  | Chuska Mountains | A. lasiocarpa | 12N | 689466 | 4017841 | 0.7096887 | 0.709654 | 0.000008 | English et al. 2001 |
| CKAMT-12 |  | Chuska Mountains | Pseudotsugamenziesii | 12N | 689466 | 4017841 | 0.7097131 | 0.7096784 | 0.00001 | English et al. 2001 |
| CKAMT-14 |  | Chuska Mountains | P. menziesii | 12N | 689466 | 4017841 | 0.7097982 | 0.7097635 | 0.000009 | English et al. 2001 |
| CKAMT-6y1 |  | Chuska Mountains | Piceaengelmannii | 12N | 689466 | 4017841 | 0.7091992 | 0.7091645 | 0.000016 | English et al. 2001 |
| CKAMT-7 |  | Chuska Mountains | P. engelmannii | 12N | 689466 | 4017841 | 0.7091542 | 0.7091195 | 0.000023 | English et al. 2001 |
| CKAMT-8 |  | Chuska Mountains | P. engelmannii | 12N | 689466 | 4017841 | 0.7091573 | 0.7091226 | 0.000018 | English et al. 2001 |
| CKAMT-9 |  | Chuska Mountains | P. engelmannii | 12N | 689466 | 4017841 | 0.7099322 | 0.7098975 | 0.00001 | English et al. 2001 |
| CKAMT-11y30 |  | Chuska Mountains | P. engelmannii | 12N | 689466 | 4017841 | 0.7102592 | 0.7102245 | 0.000026 | English et al. 2001 |
| CKAMT-16y1 |  | Chuska Mountains | P. engelmannii | 12N | 689466 | 4017841 | 0.7098194 | 0.7097847 | 0.000048 | English et al. 2001 |
| CKAMT-4 |  | Chuska Mountains |  | 12N | 689466 | 4017841 | 0.708707 | 0.7086723 | 0.000009 | English et al. 2001 |
| CKAMT-17 |  | Chuska Mountains |  | 12N | 689466 | 4017841 | 0.7097812 | 0.7097465 | 0.000026 | English et al. 2001 |
| CKCMT-70 |  | Chuska Mountains |  | 12N | 689466 | 4017841 | 0.7092391 | 0.7092044 | 0.000008 | English et al. 2001 |
| CKJMT-868 |  | Chuska Mountains | A. lasiocarpa | 12N | 680393 | 4016014 | 0.709534 | 0.7094993 | 0.000024 | English et al. 2001 |
| CKJMT-869 |  | Chuska Mountains | A. lasiocarpa | 12N | 680393 | 4016014 | 0.7094656 | 0.7094309 | 0.000042 | English et al. 2001 |
| CKJMT-871 |  | Chuska Mountains | A. lasiocarpa | 12N | 680393 | 4016014 | 0.7094814 | 0.7094467 | 0.00003 | English et al. 2001 |
| CKJMT-872 |  | Chuska Mountains | A. lasiocarpa | 12N | 680393 | 4016014 | 0.7095433 | 0.7095086 | 0.00001 | English et al. 2001 |
| CKJMT-878 |  | Chuska Mountains | P. engelmannii | 12N | 680393 | 4016014 | 0.7096538 | 0.7096191 | 0.000018 | English et al. 2001 |
| CKJMT-879 |  | Chuska Mountains | P. engelmannii | 12N | 680393 | 4016014 | 0.7096549 | 0.7096202 | 0.000064 | English et al. 2001 |
| CKJMT-880 |  | Chuska Mountains | P. engelmannii | 12N | 680393 | 4016014 | 0.709615 | 0.7095803 | 0.000032 | English et al. 2001 |
| CKJMT-881 |  | Chuska Mountains | P. engelmannii | 12N | 680393 | 4016014 | 0.7095553 | 0.7095206 | 0.00003 | English et al. 2001 |
| CKJMT-882 |  | Chuska Mountains | P. engelmannii | 12N | 680393 | 4016014 | 0.7094369 | 0.7094022 | 0.000031 | English et al. 2001 |
| chm-214b |  | Chuska Mountains | P. ponderosa | 12N | 689400 | 4018579 | 0.70707 | 0.70706 | 0.00002 | Reynolds et al 2005 |
| chm-214d |  | Chuska Mountains | P. ponderosa | 12N | 689400 | 4018579 | 0.70714 | 0.70713 | 0.00017 | Reynolds et al 2005 |
| chm-215a |  | Chuska Mountains | P. ponderosa | 12N | 689380 | 4018363 | 0.70915 | 0.70914 | 0.00002 | Reynolds et al 2005 |
| chm-215b |  | Chuska Mountains | P. ponderosa | 12N | 689380 | 4018363 | 0.70918 | 0.70917 | 0.00012 | Reynolds et al 2005 |
| chm-215c |  | Chuska Mountains | P. ponderosa | 12N | 689380 | 4018363 | 0.70893 | 0.70892 | 0.00019 | Reynolds et al 2005 |
| chm-215d |  | Chuska Mountains | P. ponderosa | 12N | 689380 | 4018363 | 0.70914 | 0.70913 | 0.0001 | Reynolds et al 2005 |
| chm-215e |  | Chuska Mountains | P. ponderosa | 12N | 689380 | 4018363 | 0.70941 | 0.7094 | 0.00026 | Reynolds et al 2005 |
| chm-216a |  | Chuska Mountains | P. ponderosa | 12N | 689380 | 4018363 | 0.70871 | 0.7087 | 0.00024 | Reynolds et al 2005 |
| chm-216b |  | Chuska Mountains | P. ponderosa | 12N | 689380 | 4018363 | 0.70842 | 0.70841 | 0.00011 | Reynolds et al 2005 |
| chm-216c |  | Chuska Mountains | P. ponderosa | 12N | 689380 | 4018363 | 0.70857 | 0.70856 | 0.00008 | Reynolds et al 2005 |
| chm-216d |  | Chuska Mountains | P. ponderosa | 12N | 689380 | 4018363 | 0.70927 | 0.70926 | 0.00009 | Reynolds et al 2005 |
| chm-217d |  | Chuska Mountains | P. ponderosa | 12N | 670382 | 4007149 | 0.70936 | 0.70935 | 0.00001 | Reynolds et al 2005 |
| chm-218a |  | Chuska Mountains | P. ponderosa | 12N | 677576 | 4031679 | 0.70968 | 0.70967 | 0.00013 | Reynolds et al 2005 |
| chm-218b |  | Chuska Mountains | P. ponderosa | 12N | 677576 | 4031679 | 0.70959 | 0.70958 | 0.00021 | Reynolds et al 2005 |
| chm-218c |  | Chuska Mountains | P. ponderosa | 12N | 677576 | 4031679 | 0.70956 | 0.70955 | 0.00038 | Reynolds et al 2005 |
| chm-218d |  | Chuska Mountains | P. ponderosa | 12N | 677576 | 4031679 | 0.70952 | 0.70951 | 0.00012 | Reynolds et al 2005 |
| chm-218e |  | Chuska Mountains | P. ponderosa | 12N | 677576 | 4031679 | 0.70947 | 0.70946 | 0.00011 | Reynolds et al 2005 |
| chm-219a |  | Chuska Mountains | P. ponderosa | 12N | 670472 | 4031813 | 0.70975 | 0.70974 | 0.00008 | Reynolds et al 2005 |
| chm-219b |  | Chuska Mountains | P. ponderosa | 12N | 670472 | 4031813 | 0.70951 | 0.7095 | 0.00012 | Reynolds et al 2005 |
| chm-219d |  | Chuska Mountains | P. ponderosa | 12N | 670472 | 4031813 | 0.70953 | 0.70952 | 0.00009 | Reynolds et al 2005 |
| chm-219e |  | Chuska Mountains | P. ponderosa | 12N | 670472 | 4031813 | 0.70958 | 0.70957 | 0.00016 | Reynolds et al 2005 |
| cu-226a |  | Cuba Mesa | P. ponderosa | 12N | 840807 | 3999023 | 0.71391 | 0.7139 | 0.00031 | Reynolds et al 2005 |
| cu-226b |  | Cuba Mesa | P. ponderosa | 12N | 840807 | 3999023 | 0.71432 | 0.71431 | 0.00034 | Reynolds et al 2005 |
| cu-226c |  | Cuba Mesa | P. ponderosa | 12N | 840807 | 3999023 | 0.71418 | 0.71417 | 0.00036 | Reynolds et al 2005 |
| cu-226d |  | Cuba Mesa | P. ponderosa | 12N | 840807 | 3999023 | 0.71414 | 0.71413 | 0.0001 | Reynolds et al 2005 |
| cu-226e |  | Cuba Mesa | P. ponderosa | 12N | 840807 | 3999023 | 0.71396 | 0.71395 | 0.0004 | Reynolds et al 2005 |
| hb-1-b |  | Hosta Butte | P. ponderosa | 12N | 683747 | 3948476 | 0.71077 | 0.71076 | 0.00004 | Reynolds et al 2005 |
| hb-1-c |  | Hosta Butte | P. ponderosa | 12N | 683747 | 3948476 | 0.71129 | 0.71128 | 0.00012 | Reynolds et al 2005 |
| hb-1-e |  | Hosta Butte | P. ponderosa | 12N | 683747 | 3948476 | 0.71096 | 0.71095 | 0.00008 | Reynolds et al 2005 |
| hb-1-k |  | Hosta Butte | P. ponderosa | 12N | 683747 | 3948476 | 0.71052 | 0.71051 | 0.00007 | Reynolds et al 2005 |
| hb-2 |  | Hosta Butte | P. ponderosa | 12N | 683747 | 3948476 | 0.71128 | 0.71127 | 0.00011 | Reynolds et al 2005 |
| lpm-220a |  | La Plata Mountains | P. ponderosa | 12N | 762373 | 4135842 | 0.71072 | 0.71071 | 0.00019 | Reynolds et al 2005 |
| lpm-220b |  | La Plata Mountains | P. ponderosa | 12N | 762373 | 4135842 | 0.71126 | 0.71125 | 0.00015 | Reynolds et al 2005 |
| lpm-220c |  | La Plata Mountains | P. ponderosa | 12N | 762373 | 4135842 | 0.70986 | 0.70985 | 0.00006 | Reynolds et al 2005 |
| lpm-220d |  | La Plata Mountains | P. ponderosa | 12N | 762373 | 4135842 | 0.71038 | 0.71037 | 0.00005 | Reynolds et al 2005 |
| lpm-220e |  | La Plata Mountains | P. ponderosa | 12N | 762373 | 4135842 | 0.71068 | 0.71067 | 0.00009 | Reynolds et al 2005 |
| lpm-222a |  | La Plata Mountains | Abies engelmanii | 12N | 778795 | 4155447 | 0.70948 | 0.70947 | 0.00014 | Reynolds et al 2005 |
| lpm-222b |  | La Plata Mountains | Populus tremuloides | 12N | 778795 | 4155447 | 0.70955 | 0.70954 | 0.00004 | Reynolds et al 2005 |
| lpm-222c |  | La Plata Mountains | Populus tremuloides | 12N | 778795 | 4155447 | 0.70965 | 0.70964 | 0.00039 | Reynolds et al 2005 |
| lpm-222b |  | La Plata Mountains | Abies lasiocarpa | 12N | 778795 | 4155447 | 0.70866 | 0.70865 | 0.00019 | Reynolds et al 2005 |
| lpm-223a |  | La Plata Mountains | P. ponderosa | 12N | 762270 | 4160988 | 0.7094 | 0.70939 | 0.00016 | Reynolds et al 2005 |
| lpm-223a |  | La Plata Mountains | P. ponderosa | 12N | 762270 | 4160988 | 0.7094 | 0.70939 | 0.00011 | Reynolds et al 2005 |
| lpm-223b |  | La Plata Mountains | P. ponderosa | 12N | 762270 | 4160988 | 0.70935 | 0.70934 | 0.00018 | Reynolds et al 2005 |
| lpm-223c |  | La Plata Mountains | P. ponderosa | 12N | 762270 | 4160988 | 0.70983 | 0.70982 | 0.00024 | Reynolds et al 2005 |
| lpm-223c |  | La Plata Mountains | P. ponderosa | 12N | 762270 | 4160988 | 0.70973 | 0.70972 | 0.0001 | Reynolds et al 2005 |
| lpm-224c |  | La Plata Mountains | P. ponderosa | 12N | 806502 | 4140338 | 0.71103 | 0.71102 | 0.00024 | Reynolds et al 2005 |
| lpm-224c |  | La Plata Mountains | P. ponderosa | 12N | 806502 | 4140338 | 0.71094 | 0.71093 | 0.00009 | Reynolds et al 2005 |
| lpm-224d |  | La Plata Mountains | P. ponderosa | 12N | 806502 | 4140338 | 0.71132 | 0.71131 | 0.00013 | Reynolds et al 2005 |
| MTA-34 |  | San Mateo Mountains | A. lasiocarpa | 12N | 785138 | 3933439 | 0.708049 | 0.7080143 | 0.000018 | English et al. 2001 |
| MTA-37 |  | San Mateo Mountains | A. lasiocarpa | 12N | 785138 | 3933439 | 0.7079724 | 0.7079377 | 0.000022 | English et al. 2001 |
| MTA-38 |  | San Mateo Mountains | A. lasiocarpa | 12N | 785138 | 3933439 | 0.7078196 | 0.7077849 | 0.000011 | English et al. 2001 |
| MTA-41 |  | San Mateo Mountains | A. lasiocarpa | 12N | 785138 | 3933439 | 0.707923 | 0.7078883 | 0.000009 | English et al. 2001 |
| MTA-43 |  | San Mateo Mountains | P. pungens | 12N | 785138 | 3933439 | 0.7077162 | 0.7076815 | 0.00001 | English et al. 2001 |
| MTA-44 |  | San Mateo Mountains | P. pungens | 12N | 785138 | 3933439 | 0.7079408 | 0.7079061 | 0.000045 | English et al. 2001 |
| MTA-45 |  | San Mateo Mountains | P. pungens | 12N | 785138 | 3933439 | 0.7079899 | 0.7079552 | 0.000011 | English et al. 2001 |
| MTA-46 |  | San Mateo Mountains | P. pungens | 12N | 785138 | 3933439 | 0.70799 | 0.7079553 | 0.000012 | English et al. 2001 |
| MTA-47 |  | San Mateo Mountains | P. pungens | 12N | 785138 | 3933439 | 0.7079763 | 0.7079416 | 0.000009 | English et al. 2001 |
| MTA-33 |  | San Mateo Mountains | P. engelmannii | 12N | 785138 | 3933439 | 0.7079068 | 0.7078721 | 0.00002 | English et al. 2001 |
| MTA-35 |  | San Mateo Mountains | P. engelmannii | 12N | 785138 | 3933439 | 0.708185 | 0.7081503 | 0.00001 | English et al. 2001 |
| MTA-36 |  | San Mateo Mountains | P. engelmannii | 12N | 785138 | 3933439 | 0.7078218 | 0.7077871 | 0.000009 | English et al. 2001 |
| MTA-39 |  | San Mateo Mountains | P. engelmannii | 12N | 785138 | 3933439 | 0.7076142 | 0.7075795 | 0.000009 | English et al. 2001 |
| MTA-40 |  | San Mateo Mountains | P. engelmannii | 12N | 785138 | 3933439 | 0.7076131 | 0.7075784 | 0.00001 | English et al. 2001 |
| MTA-32 |  | San Mateo Mountains |  | 12N | 785138 | 3933439 | 0.7075694 | 0.7075347 | 0.00001 | English et al. 2001 |
| MTB-55 |  | San Mateo Mountains | A. lasiocarpa | 12N | 801773 | 3909175 | 0.7081688 | 0.7081341 | 0.000016 | English et al. 2001 |
| MTB-56 |  | San Mateo Mountains | A. lasiocarpa | 12N | 801773 | 3909175 | 0.707668 | 0.7076333 | 0.00002 | English et al. 2001 |
| MTB-58 |  | San Mateo Mountains | A. lasiocarpa | 12N | 801773 | 3909175 | 0.7086035 | 0.7085688 | 0.000028 | English et al. 2001 |
| MTB-59 |  | San Mateo Mountains | A. lasiocarpa | 12N | 801773 | 3909175 | 0.7083906 | 0.7083559 | 0.00006 | English et al. 2001 |
| MTB-60 |  | San Mateo Mountains | P. pungens | 12N | 801773 | 3909175 | 0.706826 | 0.7067913 | 0.000011 | English et al. 2001 |
| MTB-61 |  | San Mateo Mountains | P. pungens | 12N | 801773 | 3909175 | 0.7074346 | 0.7073999 | 0.00001 | English et al. 2001 |
| MTB-62 |  | San Mateo Mountains | P. pungens | 12N | 801773 | 3909175 | 0.7071347 | 0.7071 | 0 | English et al. 2001 |
| MTB-63 |  | San Mateo Mountains | P. pungens | 12N | 801773 | 3909175 | 0.7069121 | 0.7068774 | 0.000011 | English et al. 2001 |
| MTB-64 |  | San Mateo Mountains | P. pungens | 12N | 801773 | 3909175 | 0.7069395 | 0.7069048 | 0.000012 | English et al. 2001 |
| MTB-50 |  | San Mateo Mountains | P. engelmannii | 12N | 801773 | 3909175 | 0.7079545 | 0.7079198 | 0.000015 | English et al. 2001 |
| MTB-51 |  | San Mateo Mountains | P. engelmannii | 12N | 801773 | 3909175 | 0.7075538 | 0.7075191 | 0.000037 | English et al. 2001 |
| MTB-52 |  | San Mateo Mountains | P. engelmannii | 12N | 801773 | 3909175 | 0.7078085 | 0.7077738 | 0.000008 | English et al. 2001 |
| MTB-53 |  | San Mateo Mountains | P. engelmannii | 12N | 801773 | 3909175 | 0.7087104 | 0.7086757 | 0.00001 | English et al. 2001 |
| MTB-54 |  | San Mateo Mountains | P. engelmannii | 12N | 801773 | 3909175 | 0.7086832 | 0.7086485 | 0.000014 | English et al. 2001 |
| sm-208a |  | San Mateo Mountains | P. ponderosa | 12N | 782354 | 3929710 | 0.70597 | 0.70596 | 0.00006 | Reynolds et al 2005 |
| sm-208d |  | San Mateo Mountains | P. ponderosa | 12N | 782354 | 3929710 | 0.70602 | 0.70601 | 0.00009 | Reynolds et al 2005 |
| sm-209a |  | San Mateo Mountains | P. ponderosa | 12N | 804411 | 3913492 | 0.70775 | 0.70774 | 0.00015 | Reynolds et al 2005 |
| sm-209b |  | San Mateo Mountains | P. ponderosa | 12N | 804411 | 3913492 | 0.7078 | 0.70779 | 0.00027 | Reynolds et al 2005 |
| sm-209c |  | San Mateo Mountains | P. ponderosa | 12N | 804411 | 3913492 | 0.7082 | 0.70819 | 0.00007 | Reynolds et al 2005 |
| sm-209d |  | San Mateo Mountains | P. ponderosa | 12N | 804411 | 3913492 | 0.70769 | 0.70768 | 0.00009 | Reynolds et al 2005 |
| sm-210b |  | San Mateo Mountains | P. ponderosa | 12N | 796391 | 3941026 | 0.70551 | 0.7055 | 0.00005 | Reynolds et al 2005 |
| sm-210c |  | San Mateo Mountains | P. ponderosa | 12N | 796391 | 3941026 | 0.70745 | 0.70744 | 0.00009 | Reynolds et al 2005 |
| sm-210d |  | San Mateo Mountains | P. ponderosa | 12N | 796391 | 3941026 | 0.70746 | 0.70745 | 0.00018 | Reynolds et al 2005 |
| sm-210e |  | San Mateo Mountains | P. ponderosa | 12N | 796391 | 3941026 | 0.70713 | 0.70712 | 0.00008 | Reynolds et al 2005 |
| sm-211a |  | San Mateo Mountains | P. ponderosa | 12N | 796391 | 3941057 | 0.70687 | 0.70686 | 0.00008 | Reynolds et al 2005 |
| sm-211b |  | San Mateo Mountains | P. ponderosa | 12N | 796391 | 3941057 | 0.70699 | 0.70698 | 0.0001 | Reynolds et al 2005 |
| sm-211d |  | San Mateo Mountains | P. ponderosa | 12N | 796391 | 3941057 | 0.70856 | 0.70855 | 0.00024 | Reynolds et al 2005 |
| sm-212b |  | San Mateo Mountains | P. ponderosa | 12N | 790608 | 3912542 | 0.70703 | 0.70702 | 0.00004 | Reynolds et al 2005 |
| sm-212c |  | San Mateo Mountains | P. ponderosa | 12N | 790608 | 3912542 | 0.70714 | 0.70713 | 0.00004 | Reynolds et al 2005 |
| sm-212e |  | San Mateo Mountains | P. ponderosa | 12N | 790608 | 3912542 | 0.70681 | 0.7068 | 0.00008 | Reynolds et al 2005 |
| SPAMT-2 |  | San Pedro Mountains | A. concolor | 12N | 862321 | 4006806 | 0.7170741 | 0.7170394 | 0.000022 | English et al. 2001 |
| SPBMT-1 |  | San Pedro Mountains | A. concolor | 12N | 862321 | 4006806 | 0.7130604 | 0.7130257 | 0.000034 | English et al. 2001 |
| SPBMT-2 |  | San Pedro Mountains | A. concolor | 12N | 862321 | 4006806 | 0.7132927 | 0.713258 | 0.000029 | English et al. 2001 |
| SPBMT-3 |  | San Pedro Mountains | A. concolor | 12N | 862321 | 4006806 | 0.714301 | 0.7142663 | 0.00001 | English et al. 2001 |
| SPBMT-4 |  | San Pedro Mountains | A. concolor | 12N | 862321 | 4006806 | 0.7146686 | 0.7146339 | 0.000009 | English et al. 2001 |
| SPBMT-5 |  | San Pedro Mountains | A. concolor | 12N | 862321 | 4006806 | 0.7135219 | 0.7134872 | 0.000009 | English et al. 2001 |
| SPCMT-7 |  | San Pedro Mountains | A. lastocarpa | 12N | 853760 | 3992990 | 0.713674 | 0.7136393 | 0.000021 | English et al. 2001 |
| SPCMT-10 |  | San Pedro Mountains | A. lastocarpa | 12N | 853760 | 3992990 | 0.7143578 | 0.7143231 | 0.000041 | English et al. 2001 |
| SPCMT-16 |  | San Pedro Mountains | Piceapungens | 12N | 853760 | 3992990 | 0.7153053 | 0.7152706 | 0.000022 | English et al. 2001 |
| SPCMT-18 |  | San Pedro Mountains | P. pungens | 12N | 853760 | 3992990 | 0.7154763 | 0.7154416 | 0.000038 | English et al. 2001 |
| SPCMT-8 |  | San Pedro Mountains | P. engelmannii | 12N | 853760 | 3992990 | 0.7146061 | 0.7145714 | 0.000015 | English et al. 2001 |
| SPCMT-9 |  | San Pedro Mountains | P. engelmannii | 12N | 853760 | 3992990 | 0.7142536 | 0.7142189 | 0.000016 | English et al. 2001 |
| SPCMT-11 |  | San Pedro Mountains | P. engelmannii | 12N | 853760 | 3992990 | 0.7130727 | 0.713038 | 0.000021 | English et al. 2001 |
| SPCMT-12 |  | San Pedro Mountains | P. engelmannii | 12N | 853760 | 3992990 | 0.7138897 | 0.713855 | 0.000028 | English et al. 2001 |
| SPCMT-14 |  | San Pedro Mountains |  | 12N | 853760 | 3992990 | 0.7152812 | 0.7152465 | 0.000011 | English et al. 2001 |
| SPCMT-15 |  | San Pedro Mountains |  | 12N | 853760 | 3992990 | 0.715612 | 0.7155773 | 0.00002 | English et al. 2001 |
| SPDMT-19 |  | San Pedro Mountains | A. concolor | 12N | 857668 | 4004175 | 0.7130157 | 0.712981 | 0.00002 | English et al. 2001 |
| SPDMT-20 |  | San Pedro Mountains | A. concolor | 12N | 857668 | 4004175 | 0.713367 | 0.7133323 | 0.00001 | English et al. 2001 |
| SPDMT-21 |  | San Pedro Mountains | A. concolor | 12N | 857668 | 4004175 | 0.7141709 | 0.7141362 | 0.000011 | English et al. 2001 |
| SPDMT-22 |  | San Pedro Mountains | A. concolor | 12N | 857668 | 4004175 | 0.7133034 | 0.7132687 | 0.000013 | English et al. 2001 |
| SPDMT-23 |  | San Pedro Mountains | A. concolor | 12N | 857668 | 4004175 | 0.7136388 | 0.7136041 | 0.000011 | English et al. 2001 |
| SPEMT-28 |  | San Pedro Mountains | A. lastocarpa | 12N | 858452 | 3999728 | 0.7153155 | 0.7152808 | 0.000015 | English et al. 2001 |
| SPEMT-30 |  | San Pedro Mountains | A. lastocarpa | 12N | 858452 | 3999728 | 0.7155967 | 0.715562 | 0.00002 | English et al. 2001 |
| SPEMT-31 |  | San Pedro Mountains | A. lastocarpa | 12N | 858452 | 3999728 | 0.7132415 | 0.7132068 | 0.000018 | English et al. 2001 |
| SPEMT-26 |  | San Pedro Mountains | P. engelmannii | 12N | 858452 | 3999728 | 0.7152802 | 0.7152455 | 0.000021 | English et al. 2001 |
| SPEMT-27 |  | San Pedro Mountains | P. engelmannii | 12N | 858452 | 3999728 | 0.7143573 | 0.7143226 | 0.000017 | English et al. 2001 |
| SPEMT-29 |  | San Pedro Mountains | P. engelmannii | 12N | 858452 | 3999728 | 0.7153959 | 0.7153612 | 0.000011 | English et al. 2001 |
| spm-227a |  | San Pedro Mountains | P. ponderosa | 12N | 856348 | 4007054 | 0.71804 | 0.71803 | 0.0001 | Reynolds et al 2005 |
| spm-227b |  | San Pedro Mountains | P. ponderosa | 12N | 856348 | 4007054 | 0.71902 | 0.71901 | 0.00011 | Reynolds et al 2005 |
| spm-227c |  | San Pedro Mountains | P. ponderosa | 12N | 856348 | 4007054 | 0.71919 | 0.71918 | 0.00004 | Reynolds et al 2005 |
| spm-227d |  | San Pedro Mountains | P. ponderosa | 12N | 856348 | 4007054 | 0.71558 | 0.71557 | 0.00011 | Reynolds et al 2005 |
| spm-227e |  | San Pedro Mountains | P. ponderosa | 12N | 856348 | 4007054 | 0.71614 | 0.71613 | 0.0006 | Reynolds et al 2005 |
