# Supplementary material for: Strontium Isotopes and the Reconstruction of the Chaco Regional System: Evaluating Uncertainty with Bayesian Mixing Models
Source: PLoS One. 2014 May 22;9(5):e95580. doi: 10.1371/journal.pone.0095580 (PMC4031078; doi:10.1371/journal.pone.0095580)
Supplement: Table S11 — Maize Strontium Isotope Data. (DOC) [file pone.0095580.s021.doc]

| Sample site/no. | Year | Time | Provenience | 87Sr/86Sr | Error (2 SD) | Citation |
| --- | --- | --- | --- | --- | --- | --- |
| CHCU43684-4 | 1107 | Pre 1140 Maize | Gallo Cliff Dwelling | 0.70977 | 0.000011 | Benson et al 2009 |
| CHCU43684A | 1066 | Pre 1140 Maize | Gallo Cliff Dwelling | 0.71155 | 0.00016 | Benson et al 2010 |
| H10648 | 998 | Pre 1140 Maize | Pueblo Bonito Corn | 0.709892 | 0.000016 | Benson et al 2009 |
| H242/244A | 963 | Pre 1140 Maize | Pueblo Bonito Corn | 0.709319 | 0.000016 | Benson et al 2009 |
| H242/244B | 960 | Pre 1140 Maize | Pueblo Bonito Corn | 0.709475 | 0.000042 | Benson et al 2009 |
| H254/258B | 955 | Pre 1140 Maize | Pueblo Bonito Corn | 0.709225 | 0.000018 | Benson et al 2009 |
| H254/258C | 834 | Pre 1140 Maize | Pueblo Bonito Corn | 0.70928 | 0.000014 | Benson et al 2009 |
| CHCU32288-1 | 1184 | Post 1140 Maize | Chetro Ketl 92 | 0.709523 | 0.000014 | Benson et al 2009 |
| CHCU32288-2 | 1186 | Post 1140 Maize | Chetro Ketl 92 | 0.70935 | 0.000009 | Benson et al 2009 |
| CHCU32289-1 | 1186 | Post 1140 Maize | Chetro Ketl 92 | 0.709547 | 0.0000015 | Benson et al 2009 |
| CHCU32299-1 | 1240 | Post 1140 Maize | Kin Kletso | 0.710747 | 0.00001 | Benson et al 2009 |
| CHCU32999-1 | 1206 | Post 1140 Maize | Kin Kletso | 0.709258 | 0.000015 | Benson et al 2009 |
| CHCU32999-2 | 1184 | Post 1140 Maize | Kin Kletso | 0.70928 | 0.000012 | Benson et al 2009 |
| CHCU43684-1 | 1208 | Post 1140 Maize | Gallo Cliff Dwelling | 0.71088 | 0.000014 | Benson et al 2009 |
| CHCU43684-10 | 1188 | Post 1140 Maize | Gallo Cliff Dwelling | 0.709961 | 0.000014 | Benson et al 2009 |
| CHCU43684-11 | 1188 | Post 1140 Maize | Gallo Cliff Dwelling | 0.71027 | 0.000013 | Benson et al 2009 |
| CHCU43684-12 | 1184 | Post 1140 Maize | Gallo Cliff Dwelling | 0.710198 | 0.00001 | Benson et al 2009 |
| CHCU43684-13 | 1185 | Post 1140 Maize | Gallo Cliff Dwelling | 0.71158 | 0.000013 | Benson et al 2009 |
| CHCU43684-14 | 1186 | Post 1140 Maize | Gallo Cliff Dwelling | 0.711575 | 0.000015 | Benson et al 2009 |
| CHCU43684-15 | 1185 | Post 1140 Maize | Gallo Cliff Dwelling | 0.710143 | 0.000015 | Benson et al 2009 |
| CHCU43684-16 | 1182 | Post 1140 Maize | Gallo Cliff Dwelling | 0.710094 | 0.000013 | Benson et al 2009 |
| CHCU43684-2 | 1194 | Post 1140 Maize | Gallo Cliff Dwelling | 0.711062 | 0.000014 | Benson et al 2009 |
| CHCU43684-3 | 1206 | Post 1140 Maize | Gallo Cliff Dwelling | 0.709638 | 0.000011 | Benson et al 2009 |
| CHCU43684-5 | 1184 | Post 1140 Maize | Gallo Cliff Dwelling | 0.709586 | 0.000015 | Benson et al 2009 |
| CHCU43684-6 | 1186 | Post 1140 Maize | Gallo Cliff Dwelling | 0.709412 | 0.000011 | Benson et al 2009 |
| CHCU43684-7 | 1184 | Post 1140 Maize | Gallo Cliff Dwelling | 0.710244 | 0.00001 | Benson et al 2009 |
| CHCU43684-8 | 1183 | Post 1140 Maize | Gallo Cliff Dwelling | 0.709579 | 0.000011 | Benson et al 2009 |
| CHCU43684-9 | 1206 | Post 1140 Maize | Gallo Cliff Dwelling | 0.71001 | 0.000014 | Benson et al 2009 |
| CHCU43684B | 1194 | Post 1140 Maize | Gallo Cliff Dwelling | 0.71 | 0.00024 | Benson et al 2010 |
| CHCU43684C | 1191 | Post 1140 Maize | Gallo Cliff Dwelling | 0.71103 | 0.00013 | Benson et al 2010 |
| CHCU43684D | 1188 | Post 1140 Maize | Gallo Cliff Dwelling | 0.71335 | 0.00005 | Benson et al 2010 |
| CHCU43684E | 1196 | Post 1140 Maize | Gallo Cliff Dwelling | 0.71467 | 0.00005 | Benson et al 2010 |
| CHCU43684F | 1194 | Post 1140 Maize | Gallo Cliff Dwelling | 0.7125 | 0.00027 | Benson et al 2010 |
| CHCU43684G | 1200 | Post 1140 Maize | Gallo Cliff Dwelling | 0.71089 | 0.0001 | Benson et al 2010 |
| CHCU43684H | 1194 | Post 1140 Maize | Gallo Cliff Dwelling | 0.71005 | 0.00005 | Benson et al 2010 |
| CHCU43684I | 1186 | Post 1140 Maize | Gallo Cliff Dwelling | 0.71302 | 0.00011 | Benson et al 2010 |
| CHCU43684J | 1188 | Post 1140 Maize | Gallo Cliff Dwelling | 0.71183 | 0.00003 | Benson et al 2010 |
| CHCU57-1 | 1211 | Post 1140 Maize | BC 236 | 0.710165 | 0.000014 | Benson et al 2009 |
| H254/258A | 1127 | Post 1140 Maize | Pueblo Bonito Corn | 0.709394 | 0.00001 | Benson et al 2009 |
| H7673 | 1213 | Post 1140 Maize | Pueblo Bonito Corn | 0.709328 | 0.000011 | Benson et al 2009 |
| CHCU2685-1 | 1756 | Historic | Cliff Face | 0.713553 | 0.0000013 | Benson et al 2009 |
| CHCU2685-2 | 1490 | Historic | Cliff Face | 0.710082 | 0.000015 | Benson et al 2009 |
| CHCU50553-1 | 1847 | Historic | Cliff Face Room 29MC499 | 0.710769 | 0.000022 | Benson et al 2009 |
| CHCU50553-2 | 1850 | Historic | Cliff Face Room 29MC499 | 0.711302 | 0.000014 | Benson et al 2009 |
| Site 1 Chaco E Cob1 | 1754 | Historic | 29SJ176, LA 40176 | 0.710134 | 0.000011 | Benson et al 2009 |
| Site 1 Chaco E Cob2 | 1756 | Historic | 29SJ176, LA 40176 | 0.709839 | 0.000016 | Benson et al 2009 |
