# Supplementary material for: Strontium Isotopes and the Reconstruction of the Chaco Regional System: Evaluating Uncertainty with Bayesian Mixing Models
Source: PLoS One. 2014 May 22;9(5):e95580. doi: 10.1371/journal.pone.0095580 (PMC4031078; doi:10.1371/journal.pone.0095580)
Supplement: Table S12 — Chaco Timber Strontium Isotope Data. (DOC) [file pone.0095580.s022.doc]

| Sample Name | Site # | Site Name | Taxa | 87 Sr/86 Sr corrected | 87 Sr/86 Sr uncorrected | Error (SD) | Citation |
| --- | --- | --- | --- | --- | --- | --- | --- |
| cnm-2079 | Pueblo Bonito | Room 171 | Pinus | 0.71177 | 0.71176 | 0.00011 | Reynolds et al 2005 |
| cnm-2636 | Pueblo Bonito | Room 299 | Pinus | 0.70985 | 0.70984 | 0.00033 | Reynolds et al 2005 |
| cnm-2642 | Pueblo Bonito | Room 300 | Pinus | 0.70969 | 0.70968 | 0.00033 | Reynolds et al 2005 |
| pb-290 | Pueblo Bonito | Room 227 | Pinus | 0.70951 | 0.7095 | 0.00009 | Reynolds et al 2005 |
| pb-294 | Pueblo Bonito | Room 227 | Pinus | 0.70959 | 0.70958 | 0.0001 | Reynolds et al 2005 |
| pb-304 | Pueblo Bonito | Room 317 | Pinus | 0.70928 | 0.70927 | 0.0001 | Reynolds et al 2005 |
| pb-34 | Pueblo Bonito | Room 320 | Pinus | 0.70954 | 0.70953 | 0.00003 | Reynolds et al 2005 |
| pb-341 | Pueblo Bonito | Room 320 | Pinus | 0.70931 | 0.7093 | 0.0001 | Reynolds et al 2005 |
| pb-356 | Pueblo Bonito | Room 326 | Pinus | 0.70959 | 0.70958 | 0.00023 | Reynolds et al 2005 |
| pb-512 | Pueblo Bonito | Room 209 | Pinus | 0.71009 | 0.71008 | 0.00019 | Reynolds et al 2005 |
| pb-532 | Pueblo Bonito | Room 242 | Pinus | 0.70931 | 0.7093 | 0.00007 | Reynolds et al 2005 |
| pb-540 | Pueblo Bonito | Room 244 | Pinus | 0.71035 | 0.71034 | 0.00011 | Reynolds et al 2005 |
| pb-577 | Pueblo Bonito | Room 299 | Pinus | 0.70985 | 0.70984 | 0.0001 | Reynolds et al 2005 |
| pb-581 | Pueblo Bonito | Room 299 | Pinus | 0.7099 | 0.70989 | 0.00058 | Reynolds et al 2005 |
| ckk-40 nps | Pueblo Bonito | Room 7 (KK) | Pinus | 0.71003 | 0.71002 | 0.00011 | Reynolds et al 2005 |
| ckk-41 nps | Pueblo Bonito | Room 7 (KK) | Pinus | 0.71001 | 0.71 | 0.00008 | Reynolds et al 2005 |
| ckk-72 nps | Pueblo Bonito | Room 25 (KK) | Pinus | 0.71575 | 0.71574 | 0.00014 | Reynolds et al 2005 |
| cnm-3338 nps | Pueblo Bonito | Kiva G (PB) | Pinus | 0.70931 | 0.7093 | 0.00007 | Reynolds et al 2005 |
| cnm-3453 nps | Pueblo Bonito | Room 49 (KK) | Pinus | 0.70865 | 0.70864 | 0.00011 | Reynolds et al 2005 |
| cnm-3455 nps | Pueblo Bonito | Room 49 (KK) | Pinus | 0.7095 | 0.70949 | 0.00028 | Reynolds et al 2005 |
| ck-1165 | Chetro Ketl | Room 46/48 | Pinus | 0.70941 | 0.7094 | 0.00011 | Reynolds et al 2005 |
| ck-1224 | Chetro Ketl | Room 39A | Pinus | 0.70961 | 0.7096 | 0.00033 | Reynolds et al 2005 |
| ck-1225 | Chetro Ketl | Room 39A | Pinus | 0.71069 | 0.71068 | 0.00008 | Reynolds et al 2005 |
| ck-1226 | Chetro Ketl | Room 39A | Pinus | 0.7095 | 0.70949 | 0.00005 | Reynolds et al 2005 |
| ck-1232 | Chetro Ketl | Room 39A | Pinus | 0.70941 | 0.7094 | 0.00013 | Reynolds et al 2005 |
| ck-1233 | Chetro Ketl | Room 39A | Pinus | 0.70954 | 0.70953 | 0.00024 | Reynolds et al 2005 |
| ck-1239 | Chetro Ketl | Room 39A | Pinus | 0.71103 | 0.71102 | 0.00007 | Reynolds et al 2005 |
| ck-1275 | Chetro Ketl | Room 92 | Pinus | 0.70929 | 0.70928 | 0.00006 | Reynolds et al 2005 |
| ck-1300 | Chetro Ketl | Room 106 | Pinus | 0.71698 | 0.71697 | 0.00022 | Reynolds et al 2005 |
| ck-1303 | Chetro Ketl | Room 106 | Pinus | 0.7092 | 0.70919 | 0.00012 | Reynolds et al 2005 |
| ck-1307 | Chetro Ketl | Room 106 | Pinus | 0.71029 | 0.71028 | 0.00035 | Reynolds et al 2005 |
| cnm-4133 | Chetro Ketl | Room 114 | Pinus | 0.71019 | 0.71018 | 0.00025 | Reynolds et al 2005 |
| cnm-4191 | Chetro Ketl | Room 119 | Pinus | 0.71002 | 0.71001 | 0.00042 | Reynolds et al 2005 |
| cnm-4192 | Chetro Ketl | Room 119 | Pinus | 0.70973 | 0.70972 | 0.00012 | Reynolds et al 2005 |
| cnm-2504 | Chetro Ketl | Room 40 (PdA) | Pinus | 0.70958 | 0.70957 | 0.00036 | Reynolds et al 2005 |
| ck-1144 nps | Chetro Ketl | Room 43 (CK) | Pinus | 0.70952 | 0.70951 | 0.00024 | Reynolds et al 2005 |
| cnm-1492 nps | Chetro Ketl | Room 37 (PdA) | Pinus | 0.70917 | 0.70916 | 0.0001 | Reynolds et al 2005 |
| cnm-1617 nps | Chetro Ketl | Room 87 (PdA) | Pinus | 0.70961 | 0.7096 | 0.00047 | Reynolds et al 2005 |
| cnm-1678 nps | Chetro Ketl | Room 144 (PdA) | Pinus | 0.70979 | 0.70978 | 0.00028 | Reynolds et al 2005 |
| cnm-1186 | Pueblo del Arroyo | Room 43 | Pinus | 0.71097 | 0.71096 | 0.00026 | Reynolds et al 2005 |
| cnm-1187 | Pueblo del Arroyo | Room 43 | Pinus | 0.71012 | 0.71011 | 0.00022 | Reynolds et al 2005 |
| cnm-1188 | Pueblo del Arroyo | Room 43 | Pinus | 0.70998 | 0.70997 | 0.00008 | Reynolds et al 2005 |
| cnm-1363 | Pueblo del Arroyo | Room 8 | Pinus | 0.70918 | 0.70917 | 0.00025 | Reynolds et al 2005 |
| cnm-1377 | Pueblo del Arroyo | Room 9A III | Pinus | 0.71017 | 0.71016 | 0.00019 | Reynolds et al 2005 |
| cnm-1390 | Pueblo del Arroyo | Room 9A III | Pinus | 0.7084 | 0.70839 | 0.00016 | Reynolds et al 2005 |
| cnm-1392 | Pueblo del Arroyo | Room 9A III | Pinus | 0.70947 | 0.70946 | 0.00014 | Reynolds et al 2005 |
| cnm-1393 | Pueblo del Arroyo | Room 9A | Pinus | 0.70982 | 0.70981 | 0.00027 | Reynolds et al 2005 |
| cnm-1405 | Pueblo del Arroyo | Room 9A III | Pinus | 0.70958 | 0.70957 | 0.0001 | Reynolds et al 2005 |
| cnm-1649 | Pueblo del Arroyo | Room 102 | Pinus | 0.7095 | 0.70949 | 0.00006 | Reynolds et al 2005 |
| cnm-2525 | Pueblo del Arroyo | Room 43 | Pinus | 0.71044 | 0.71043 | 0.00011 | Reynolds et al 2005 |
| cnm-3408 | Pueblo del Arroyo | Room 95 | Pinus | 0.7092 | 0.70919 | 0.00019 | Reynolds et al 2005 |
| cnm-2310 | Pueblo del Arroyo | Room 8 | Abies | 0.7092612 | 0.7092265 | 0.000008 | English et al 2001 |
| cnm-2500 | Pueblo del Arroyo | Room 37 | Abies | 0.7099992 | 0.7099645 | 0.000021 | English et al 2001 |
| cnm-2521 | Pueblo del Arroyo | Room 43 | Abies | 0.7097413 | 0.7097066 | 0.000021 | English et al 2001 |
| cnm-479 | Pueblo del Arroyo | Room 8 | Abies | 0.7095292 | 0.7094945 | 0.00001 | English et al 2001 |
| jpb-132 | Pueblo del Arroyo | Room 46 | Abies | 0.7091934 | 0.7091587 | 0.000009 | English et al 2001 |
| cnm-1398 | Pueblo del Arroyo | Room 9A | Abies | 0.7096796 | 0.7096449 | 0.000012 | English et al 2001 |
| cnm-1033 | Pueblo del Arroyo | Room 13 | Abies | 0.7093391 | 0.7093044 | 0.000009 | English et al 2001 |
| cnm 1036 | Pueblo del Arroyo | Room 13 | Abies | 0.70985 | 0.7098153 | 0.000012 | English et al 2001 |
| cnm-1832 | Pueblo del Arroyo | Room 8 | Abies | 0.709592 | 0.7095573 | 0.000013 | English et al 2001 |
| cnm-23539 | Pueblo del Arroyo | Room 53 | Picea | 0.70857 | 0.7085353 | 0.000013 | English et al 2001 |
| cnm-1481 | Pueblo del Arroyo | Room 34 | Picea | 0.7084334 | 0.7083987 | 0.000024 | English et al 2001 |
| cnm-1605 | Pueblo del Arroyo | Room 62 | Picea | 0.7092046 | 0.7091699 | 0.000009 | English et al 2001 |
| p8-436 | Pueblo Bonito | Room 86 | Abies | 0.7085922 | 0.7085575 | 0.000009 | English et al 2001 |
| p8-441 | Pueblo Bonito | Room 86 | Abies | 0.7098005 | 0.7097658 | 0.000013 | English et al 2001 |
| p8-442 | Pueblo Bonito | Room 86 | Abies | 0.7097959 | 0.7097612 | 0.000011 | English et al 2001 |
| p8-585 | Pueblo Bonito | Room 299 | Abies | 0.7094707 | 0.709436 | 0.000016 | English et al 2001 |
| cnm-2188 | Pueblo Bonito | Room 247 | Abies | 0.7093993 | 0.7093646 | 0.000009 | English et al 2001 |
| cnm-3967 | Pueblo Bonito | Room 148 | Abies | 0.7090319 | 0.7089972 | 0.000026 | English et al 2001 |
| cnm-3969 | Pueblo Bonito | Room 148 | Abies | 0.7096244 | 0.7095897 | 0.000019 | English et al 2001 |
| pb-5666 | Pueblo Bonito | Room 295 | Abies | 0.706452 | 0.7064173 | 0.000009 | English et al 2001 |
| pb-727 | Pueblo Bonito | Room 100(?) | Abies | 0.7098056 | 0.7097709 | 0.000024 | English et al 2001 |
| p8-459 | Pueblo Bonito | Room 100A | Abies | 0.7097331 | 0.7096984 | 0.000009 | English et al 2001 |
| pb-445 | Pueblo Bonito | Room 89 | Abies | 0.7042162 | 0.7041815 | 0.00001 | English et al 2001 |
| pb-452 | Pueblo Bonito | Room 93 | Abies | 0.7085748 | 0.7085401 | 0.000011 | English et al 2001 |
| cnm-970 | Pueblo Bonito | Room 148 | Abies | 0.7095998 | 0.7095651 | 0.000016 | English et al 2001 |
| pb-799 | Pueblo Bonito | Room 171 | Abies | 0.707522 | 0.7074873 | 0.00001 | English et al 2001 |
| pb-869 | Pueblo Bonito | Kiva D | Abies | 0.7090029 | 0.7089682 | 0.000014 | English et al 2001 |
| pb-871 | Pueblo Bonito |  | Abies | 0.70906 | 0.7090253 | 0.000009 | English et al 2001 |
| pb-567 | Pueblo Bonito | Room 295 | Picea | 0.7079422 | 0.7079075 | 0.000011 | English et al 2001 |
| gp-2310 | Pueblo Bonito | Room 105 | Picea | 0.708218 | 0.7081833 | 0.000008 | English et al 2001 |
| pb-118 | Pueblo Bonito | 3C/III | Picea | 0.709987 | 0.7099523 | 0.000035 | English et al 2001 |
| ck-1294 | Chetro Ketl | Room 106 | Abies | 0.7096545 | 0.7096198 | 0.000028 | English et al 2001 |
| ck-1292 | Chetro Ketl | Room 106 | Abies | 0.7095788 | 0.7095441 | 0.000011 | English et al 2001 |
| ck-123 | Chetro Ketl | Room 44 | Abies | 0.7079819 | 0.7079472 | 0.000035 | English et al 2001 |
| ck-119 | Chetro Ketl | Room 44 | Abies | 0.7093431 | 0.7093084 | 0.000007 | English et al 2001 |
| cnm-3797 | Chetro Ketl | Room 89 | Abies | 0.7095991 | 0.7095644 | 0.00001 | English et al 2001 |
| ck-117 | Chetro Ketl | Room 44 | Abies | 0.7083462 | 0.7083115 | 0.000023 | English et al 2001 |
| ck-60 | Chetro Ketl | Kiva G | Abies | 0.7093729 | 0.7093382 | 0.000011 | English et al 2001 |
| cnm-2664 | Chetro Ketl | Room 62 | Picea | 0.709151 | 0.7091163 | 0.000009 | English et al 2001 |
| ck-136 | Chetro Ketl | Kiva G | Picea | 0.7087178 | 0.7086831 | 0.00003 | English et al 2001 |
| ck-72 | Chetro Ketl | Kiva G | Picea | 0.7086782 | 0.7086435 | 0.00001 | English et al 2001 |
| ck-1215 | Chetro Ketl | Room 70 | Picea | 0.7090239 | 0.7089892 | 0.000013 | English et al 2001 |
| cnm-2700 | Chetro Ketl | Room 88 | Picea | 0.7080036 | 0.7079689 | 0.000012 | English et al 2001 |
| ck-168 | Chetro Ketl | Kiva G | Picea | 0.709129 | 0.7090943 | 0.00001 | English et al 2001 |
| ck-309 | Chetro Ketl | Room 27 | Picea | 0.7079204 | 0.7078857 | 0.000016 | English et al 2001 |
| ck-319 | Chetro Ketl | Kiva N | Picea | 0.7077642 | 0.7077295 | 0.000009 | English et al 2001 |
